# Highly efficient gene inactivation by adenoviral CRISPR/Cas9 in human primary cells

**DOI:** 10.1371/journal.pone.0182974

**Published:** 2017-08-11

**Authors:** Olaf Voets, Frans Tielen, Edo Elstak, Julian Benschop, Max Grimbergen, Jan Stallen, Richard Janssen, Andre van Marle, Christian Essrich

**Affiliations:** Galapagos BV, CL Leiden, The Netherlands; University of Florida, UNITED STATES

## Abstract

Phenotypic assays using human primary cells are highly valuable tools for target discovery and validation in drug discovery. Expression knockdown (KD) of such targets in these assays allows the investigation of their role in models of disease processes. Therefore, efficient and fast modes of protein KD in phenotypic assays are required. The CRISPR/Cas9 system has been shown to be a versatile and efficient means of gene inactivation in immortalized cell lines. Here we describe the use of adenoviral (AdV) CRISPR/Cas9 vectors for efficient gene inactivation in two human primary cell types, normal human lung fibroblasts and human bronchial epithelial cells. The effects of gene inactivation were studied in the TGF-β-induced fibroblast to myofibroblast transition assay (FMT) and the epithelial to mesenchymal transition assay (EMT), which are SMAD3 dependent and reflect pathogenic mechanisms observed in fibrosis. Co-transduction (co-TD) of AdV Cas9 with SMAD3-targeting guide RNAs (gRNAs) resulted in fast and efficient genome editing judged by insertion/deletion (indel) formation, as well as significant reduction of SMAD3 protein expression and nuclear translocation. This led to phenotypic changes downstream of SMAD3 inhibition, including substantially decreased alpha smooth muscle actin and fibronectin 1 expression, which are markers for FMT and EMT, respectively. A direct comparison between co-TD of separate Cas9 and gRNA AdV, versus TD with a single “all-in-one” Cas9/gRNA AdV, revealed that both methods achieve similar levels of indel formation. These data demonstrate that AdV CRISPR/Cas9 is a useful and efficient tool for protein KD in human primary cell phenotypic assays. The use of AdV CRISPR/Cas9 may offer significant advantages over the current existing tools and should enhance target discovery and validation opportunities.

## Introduction

First-in-class drug discovery generally starts with the identification of novel targets in a relevant disease model, by pinpointing which genes contribute to a specific biological process or disease [1]. Phenotypic assays with human primary cells in combination with reduction of gene expression ("knockdown", KD) are a valuable tool to explore the function of targets in this context. Until recently, such expression KD has been largely achieved using RNA interference (RNAi) [2]. In this case, messenger RNA (mRNA) is cleaved and degraded in a sequence-specific manner, dependent on the presence of double stranded RNA molecules, such as small interfering RNAs (siRNAs) or short hairpin RNAs (shRNAs). As a result, mRNA levels of a given gene, and subsequently protein expression are decreased. Although RNAi has proven to be a highly useful technology, downregulation of unintended targets (“off-targets”) is a genuine problem, potentially resulting in unspecific effects [3]. Furthermore, using RNAi the degree of mRNA KD can vary greatly and will never reach full penetrance. While this may not pose a problem as long as the KD is sufficient to cause a change in phenotype, sometimes complete KD is required and often desirable [4]. This is generally achieved through gene knockout (KO) leading to total absence of a functional protein.

Traditional methods to generate targeted gene KOs are tedious and relatively inefficient, depending on homologous recombination (HR) of a donor sequence with the host genome. Recombination frequencies can be improved by orders of magnitude using so-called engineered nucleases, including zinc-finger nucleases (ZFNs) and transcription activator-like effector nucleases (TALENs), as well as RNA-guided endonucleases (RGENs) such as the CRISPR/Cas9 (clustered regulatory interspaced short palindromic repeats/CRISPR-associated) system [5]. These nucleases are characterized by their sequence-specific binding to and cleavage of genomic DNA, resulting in the formation of either a DNA single-strand or double-strand break (SSB and DSB, respectively). Both types of lesions need to be repaired in order to maintain genomic integrity. Presence of a DSB activates the cellular DNA repair machinery leading to repair by the error-prone non-homologous end joining (NHEJ) and/or homology-directed repair (HDR) pathways [6]. By providing a donor template for repair, one can modify the DNA sequence in a targeted fashion, a process referred to as genome editing. A gene KO or point mutant can thus be established in a relatively short time span.

Of all genomic engineering nucleases known to date the CRISPR/Cas9 system has become the most attractive, owing to its simplicity and ease of use. CRISPR/Cas9 was originally identified as part of the adaptive immune system in prokaryotes, silencing invading foreign nucleic acids, such as viruses and plasmids [7]. Recently this system has been adapted for use in eukaryotes, to allow easy manipulation of the genome. It requires two components for proper functioning: the Cas9 endonuclease and a guide RNA (gRNA) containing a targeting sequence of roughly 20 base pairs (bp) complementary to a specific genomic region [8]. By expressing these components in mammalian cells together it appears that virtually any genomic sequence can be modified. One restriction of the CRISPR/Cas9 system is the requirement of a protospacer adjacent motif (PAM), a short stretch of nucleotides present in the genomic target region immediately downstream of the gRNA binding site [9]. The nature of this PAM sequence appears to be dictated by the Cas9 orthologue used, where the most commonly used Cas9 endonuclease from *Streptococcus pyogenes* requires an NGG motif for highest cleavage efficiency. In the human genome this particular sequence is expected to statistically occur every 42 bases, which makes it likely that each gene can in principle be targeted by CRISPR/Cas9.

Among other methods, introduction of the CRISPR/Cas9 system into cells may be achieved by transfection of plasmids encoding Cas9 and gRNA, or by transduction (TD) with viral particles containing both components. Viral TD is typically more efficient compared to transfection and applicable to many cell types including human primary cells, making it suitable for screening drug targets in a relevant setup. Although lentiviruses (LVs) have been used in most cases so far as viral vectors for CRISPR/Cas9 [10,11], other viruses like adenoviruses (AdVs) may provide higher specificity and efficiency [12]. This is especially important for gene-modifications introducing a DNA-sequence ("tag") or point mutation [13,14]. So far most of the published work reporting on the CRISPR/Cas9 technology for genome editing has relied on the use of immortalized cells, which have the advantage of allowing for easy selection strategies and cloning of edited cells. However, these cells are generally very different from their original source (i.e. primary cells), due to their transformation status. So far only few studies have applied CRISPR/Cas9 to human primary cells, most likely in part because of their limited lifespan, which is restrictive for selection and enrichment strategies. Nevertheless, successful gene editing in human primary cells without drug selection has been reported in cell types such as CD4+ T-lymphocytes, dermal fibroblasts, (pre)adipocytes, endothelial cells, and airway epithelial cells [15–19]. It is likely that this can be extended to other human primary cell types as well. However, for beneficial use of genomic editing in short-lived phenotypic assays for drug discovery with primary cells, the method needs to be comparably fast, efficient and specific as RNAi.

To assess the use of an AdV-based CRISPR/Cas9 platform for target discovery and/or validation, we applied AdV TD of Cas9 and gRNA components in human primary cell models of fibrosis, focusing on the transforming growth factor (TGF)-β signaling pathway. It is demonstrated that AdV delivery of Cas9 endonuclease and gRNA lead to highly efficient editing of the SMAD3 gene ("Mothers against decapentaplegic homolog 3") in both, normal human lung fibroblasts (NHLFs) and human bronchial epithelial cells (HBECs). Delivery of Cas9 and gRNA by two separate AdVs results in similar genomic editing efficiencies when compared with a single all-in-one AdV particle containing both Cas9 and gRNA. CRISPR/Cas9-mediated editing results in very effective SMAD3 protein KD and, as a consequence, inhibits fibroblast to myofibroblast transition (FMT) and epithelial to mesenchymal transition (EMT) in NHLF and HBEC primary cell phenotypic assays, respectively. This suggests that the AdV-based CRISPR/Cas9 platform is a valuable tool to study gene function in human primary cells, which may hold great promise for the identification of novel drug targets.

## Materials and methods

### Ethics statement

The research described here has been performed according to applicable Dutch national ethics regulations and was conducted within Galapagos B.V. Galapagos’ scientists are qualified to perform research with these human materials and Galapagos makes available appropriate facilities and equipment to allow such scientists to comply with applicable laws, regulations and internal rules applicable to the use, handling and storage of the material. The human material was obtained from Epithelix Sàrl (Switzerland) and from Tissue Solutions Ltd (Scotland). Both suppliers have confirmed to Galapagos that they received informed consent from the donors to use the material for research purposes. The genetic modification of these cells as described was conducted according to permits from the Dutch authorities (reference number IG-16-019_IIk). The cells were used exclusively for target and drug discovery and were not used for human experimentation or therapy. All material is and will remain anonymized at all times.

### Cells and phenotypic assays

NHLFs were acquired from Epithelix Sàrl, Switzerland. HBECs were obtained from lung and bronchial resection tissue of patients diagnosed with idiopathic pulmonary fibrosis undergoing surgery for lung transplantation (Tissue Solutions Ltd, Scotland). HBECs were isolated by protease digestion as previously described [20]. NHLF cells were cultured in Dulbecco’s Modified Eagle Medium (DMEM, Gibco) supplemented with 10% heat-inactivated fetal bovine serum (FBS, Life Technologies), 100 units/mL of penicillin and 100 μg/mL of streptomycin (“Pen-Strep”, Gibco). For the FMT assay, NHLFs were seeded in 96 well plates coated with 3 μg/mL PureCol (Advanced Biomatrix) at a density of 3,000 cells per well and kept in DMEM supplemented with 2% FBS and Pen-Strep. Cells were transduced with AdV on day *in vitro* (DIV) 1 and refreshed on DIV 2. Cells were triggered with 2 ng/mL TGF-β1 (R&D Systems) dissolved in DMEM supplemented with 0.2% FBS and Pen-Strep on DIV 6. For the EMT assay, HBECs were cultured in Keratinocyte serum-free medium (KSFM) supplemented with 0.2 ng/mL epidermal growth factor (EGF) and 25 μg/mL bovine pituitary extract (BPE), 1 μM isoproterenol and Pen-Strep (all from Gibco). During maintenance HBECs were grown on surfaces coated with 10 μg/mL fibronectin 1 (Sigma-Aldrich), 30 μg/mL PureCol and 10 μg/mL bovine serum albumin (BSA). For the assay HBECs were seeded in 96 well plates coated with 30 μg/mL PureCol at a density of 2,500 cells per well and transduced with AdV on DIV 1, followed by refreshment on DIV 2. Triggering was performed with 0.5 ng/mL TGF-β1 and 5 ng/mL TNFα (tumor necrosis factor alpha, R&D Systems) dissolved in KSFM growth medium on DIV 6. NHLF cells and HBECs were maintained at 37°C and 5% CO_2_ and refreshed twice after thawing of the cells.

The E1- and E2A-complementing AdV packaging cell line PER.C6/E2A was cultured in DMEM supplemented with 10% FBS, 10 mM MgCl_2_ and 250 mg/mL Geneticin (Life Technologies). Geneticin was only used during the first two passages to remove PER.C6 cells without the E2A gene. PER.C6/E2A cells were sub-cultured every 3 to 4 days by seeding 10^7^ cells in a T175 flask containing 25 mL growth medium. Cells were maintained at 39°C and 10% CO_2_ and were shifted to 34°C during AdV production to ensure correct folding of their E2A-encoded temperature-sensitive DNA-binding protein.

### CRISPR/Cas9 construct design and AdV production

A human codon-optimized Cas9 based on the *S*. *pyogenes* wild-type Cas9 sequence was generated by gene synthesis (GeneArt). Guide RNA sequences were adapted from Jinek et al. 2012 [8]. The 19–20 bp target specific gRNA sequences were designed by Sigma-Aldrich. Complementary oligonucleotides containing the gRNA target sequence were ordered from Life Technologies and cloned into AdV adapter plasmids by SapI restriction enzyme digestion (Thermo Scientific). This resulted in removal of a ccdB selection cassette and was followed by ligation of the gRNA-encoding oligonucleotides using T4 DNA ligase (Thermo Scientific). Ligations were transformed into either MultiShot StripWell Mach1 (gRNA only constructs) or MAX Efficiency Stbl2 (“all-in-one” constructs) competent cells (both from Invitrogen). The AsRed reporter-gene construct was adapted from the pAsRed2-C1 vector (Clontech). For nuclear expression of the reporter a 3x NLS sequence (5’-GGAGATCCAAAAAAGAAGAGAAAGGTAGATCCAAAAAAGAAGAGAAAGGTAGATCCAAAAAAGAAGAGAAAG-GTACTCGAG-3’) was cloned directly downstream from the AsRed open reading frame. The AsRed-3xNLS sequence was cloned into an AdV adapter plasmid containing a CMV promoter by standard restriction enzyme digestion and ligation.

For the generation of AdVs, the pIPspAdapt-based constructs were transiently transfected in a 96-well format together with AdV helper DNA into the producer cells PER.C6/E2A, seeded at 25,000 cells per well [21,22]. The produced AdVs were propagated by reinfecting PER.C6/E2A seeded at a density of 50,000 cells per well. During these steps PER.C6/E2A were kept in DMEM supplemented with 10% FBS and 10 mM MgCl_2_. Titers of the crude lysates were determined as described [23].

### Adenoviral TD

Cells were transduced at a multiplicity of infection (MOI) between 2.5 and 80. The total viral load between parallel samples was kept constant at all times to ensure comparable conditions. Crude AdV lysates were diluted in the appropriate cellular growth medium, and 10–20 μL were added to each well of a 96 well plate, or 100–200 μL to each T25 flask.

### Cell viability assay

NHLFs and HBECs were seeded in 96 well plates at a density of 3,000 or 2,500 cells per well, respectively. Cells were transduced with AdV on DIV 1 and medium was refreshed on DIV 2. NHLFs and HBECs were triggered on DIV 6 with 2 ng/mL TGF-β1 or 0.5 ng/mL TGF-β1 and 5 ng/mL TNFα, respectively. Cell viability was determined on DIV 9 by adding 20 μL per well CellTiter-Blue^®^ Reagent and incubating the cells for 2 hours (HBECs) or 4 hours (NHLFs) at 37°C and 5% CO_2_ prior to measuring fluorescence on an EnVision Multilabel Plate Reader (Perkin Elmer).

### Genomic DNA isolation and SURVEYOR^®^ assay

Efficiency of CRISPR/Cas9-mediated target gene disruption was measured by using mismatch-sensitive SURVEYOR^®^ nuclease. To this end, genomic DNA from up to 10^5^ cells per sample was isolated using the ZR-96 Quick-gDNA kit (Zymo Research), followed by PCR amplification of a DNA fragment comprising the target site for the appropriate gRNA AdV construct (for sequence information see S1 Table). PCR amplification was performed using a high fidelity PCR enzyme mix (Thermo Scientific). PCR samples were cleaned up using the ZR-96 DNA Clean-up Kit (Zymo Research) and DNA was quantified on an EnVision Multilabel Plate Reader (Perkin Elmer). 400 ng PCR product per sample were annealed in NEB2 buffer (New England Biolabs) in a thermocycler and treated with SURVEYOR^®^ nuclease according to the instructions of the SURVEYOR^®^ Mutation Detection kit (Integrated DNA Technologies). DNA fragments were resolved on 1.5% agarose gels, stained with ethidium bromide and visualized with a GelDoc Universal Hood II system (BioRad). Indel percentages were calculated by densitometry of the parental and cleaved DNA fragments using the LI-COR Image Studio software as described [24].

### Immunoblotting

Primary NHLFs and HBECs were seeded at a density of 1.0x10^5^-1.5x10^5^ cells per T25 flask. Cells were transduced on DIV 1, refreshed 6–8 hours after TD and harvested on DIV 8 or DIV 12 by trypsinization. Cell pellets were lysed in lysis buffer (20 mM Tris-HCl pH 7.4, 137 mM NaCl, 1% Triton X-100, 5 mM EDTA with freshly added Halt Protease and Phosphatase Inhibitor [Thermo Scientific]) on ice for 30 minutes, followed by 10 minutes centrifugation at 20,000x g. The protein concentration of the supernatant was determined using the Pierce BCA Protein Assay Kit (Thermo Scientific). Protein lysates were mixed with NuPAGE LDS Sample Buffer (Life Technologies) and denatured at 70°C for 10 minutes. 28–35 μg protein per sample were separated on NuPAGE Novex 4–12% Bis-Tris gels (Life Technologies) according to manufacturer’s instructions. Proteins were transferred onto 0.45 μm PVDF membranes (Millipore) and blocked for 1 hour at room temperature (RT) in 5% non-fat dry milk, dissolved in Tris-buffered saline (TBS) containing 0.1% Tween-20. Membranes were incubated overnight at 4°C with primary antibody and for 1 hour at RT with secondary antibody the following day. Membranes were scanned with an Odyssey CLX imager (LI-COR Biosciences) at 700 nm and 800 nm wavelength, and band signals were quantified using the LI-COR Image Studio software. The band signal for β-actin was used to normalize for the expression of the target protein (SMAD3). Primary antibodies: rabbit anti-SMAD3 (#9513 and #9523, Cell Signaling), mouse anti-β-actin (#3700, Cell Signaling), and mouse anti-Cas9 (#C15200203, Diagenode); all antibodies were diluted 1:1,000. Secondary antibodies: IRDye 680RD goat anti-mouse, IRDye 800CW goat anti-mouse, IRDye 680RD goat anti-rabbit and IRDye 800CW goat anti-rabbit (LI-COR Biosciences) all diluted 1:10,000.

### Immunocytochemistry and high content analysis (HCA)

Cells were fixed on DIV 9 in 4% formaldehyde for 30 minutes at RT and blocked for 1 hour at RT in blocking buffer (0.2% Triton X-100, 2% FBS, 3% BSA, and 1% milk in phosphate-buffered saline [PBS]). After incubation with primary antibody at RT for 1 hour and several washes with wash buffer (PBS with 0.2% Tween-20), cells were incubated with secondary antibody at RT for 1 hour, repeatedly washed with wash buffer, and nuclei were stained with 4′,6-diamidino-2-phenyindole (DAPI) solution (Sigma-Aldrich). Primary antibodies: rabbit anti-SMAD3 (#9523, Cell Signaling) diluted 1:100, mouse anti-fibronectin 1 (#MAB1940, Merck Millipore) diluted 1:200, and mouse anti-alpha smooth muscle actin (#ab7817, Abcam) diluted 1:250. Secondary antibodies: donkey-anti-rabbit Alexa488 and donkey-anti-mouse Alexa488 (Life Technologies) both diluted 1:250.

Assay plates were imaged using the IN Cell Analyzer 6000 with a solid state laser (405, 488 nm, 561 nm, 633 nm) and a 2048x2048 pixel sCMOS camera (GE Healthcare). Fluorescent probes were imaged using the appropriate laser line and emission filter and with optimal exposure times for the best signal to noise ratios. Images were analyzed using an algorithm developed in house with the GE Developer software package. Nuclear segmentation was based on DAPI images and used for cell counting. The fibronectin 1 (FN1) or alpha smooth muscle actin (ACTA2) signals were segmented in their respective channels and intensity x area was calculated as a measure of protein expression. Target (SMAD3) protein levels were evaluated using a cytoplasmic mask based on an expanded nuclei segmentation area and intensity x area was determined. Nuclear AsRed signals were evaluated using the nuclear segmentation mask to calculate the number of AsRed positive cells.

## Results

### TD of AdV-encoded CRISPR/Cas9 components results in highly efficient genomic editing in human primary cells

**Fig 1:**
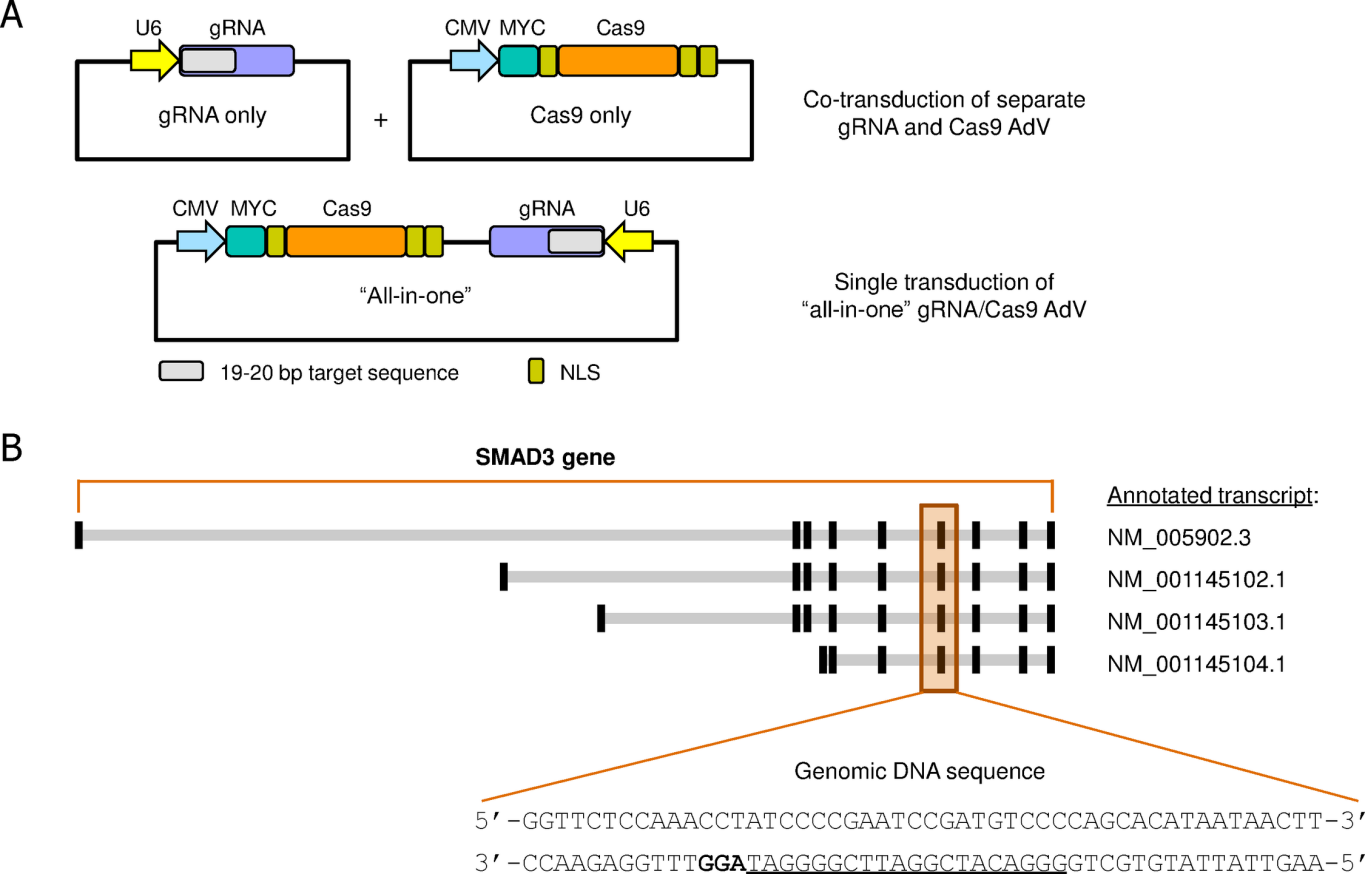
AdV CRISPR/Cas9 constructs and gRNA targeting sequences. (**A**) Overview of the AdV Cas9 and gRNA constructs used in this study. CRISPR/Cas9 AdV constructs were based on human codon optimized *S*. *pyogenes* wild type Cas9 and previously published gRNA sequences [8]. Cas9 and gRNA components were either presented in separate AdVs or together in a single AdV construct (“all-in-one”). (**B**) Genomic structure of the human SMAD3 gene with the indicated annotated transcripts. One of the gRNA sequences targeting the SMAD3 genomic DNA is underlined with the PAM in bold. NLS: nuclear localization signal.

To date most CRISPR/Cas9 studies have relied on the use of plasmid transfection or LV TD to deliver the Cas9 and gRNA components into the host, in combination with selection of recombined cell populations. Although transfection can be time-saving, it can be difficult to titrate the amount of DNA copies entering each cell. Furthermore, the efficiency of DNA transfection is often limited in primary cells and can lead to significant cell death. Delivery by LV TD may overcome these issues, but LVs can have other unwanted characteristics such as random integration into the host genome. The use of AdV may be superior in this regard as AdV has a linear double stranded genome capped with protein at its 5’ ends, which has been reported to be more specific for genome editing [12]. To test this approach, we generated AdV constructs containing either the U6 promoter-driven gRNA or CMV promoter-driven *S*. *pyogenes* wild type Cas9 endonuclease component (Fig 1A). Since both, gRNA and Cas9 protein are required for the system to work, this requires simultaneous TD of cells with the gRNA and Cas9 AdV, which may be inefficient. Therefore, we also designed an AdV construct encoding gRNA and Cas9 for expression in the same viral particle (“all-in-one”), which allows delivery by a single TD event. The constructs were designed to allow for easy exchange of the 19–20 bp target specific sequence of the gRNAs to simplify future cloning efforts for different genes of interest.

As proof of concept, the SMAD3 gene was targeted using our AdV CRISPR/Cas9 approach. SMAD3 plays an important role in TGF-β dependent signaling from the stimulated receptor (TGF-βR) at the cell surface to the nucleus, regulating transcription of various genes together with other SMAD proteins and transcription factors [25]. Triggering of the TGF-βR pathway results in increased production of extracellular matrix components as well as EMT of epithelial cells. These events are hallmarks of fibrosis. Guide RNA targeting sequences were chosen to be specific for SMAD3 and aimed at all major SMAD3 isoforms (Fig 1B and S1 Table). These 19 bp sequences were cloned into AdV adapters using standard restriction enzyme digestion and DNA ligation.

The CRISPR/Cas9 AdV constructs were tested in NHLFs and HBECs, two physiologically relevant human primary cell types, which are implicated in fibrosis *in vivo*. Initially, co-TD of these cell types with AdV encoding SMAD3-targeting or a non-targeting gRNA and Cas9 AdV was carried out. After 4–7 days the genomic DNA was analyzed for the presence of NHEJ-derived insertions or deletions (indels) in the target region, using the SURVEYOR^®^ nuclease assay. Co-TD of NHLFs (S3A Fig) or HBECs (S3B Fig) with AdV Cas9 and three different SMAD3 specific gRNAs (SMAD3_v39, _v40, _v41) led to significant levels of indel formation in the targeted region of the SMAD3 open reading frame, with SMAD3_v39 consistently showing higher mutagenesis rates compared to _v40 and _v41 in both cell types and repeat experiments (not shown). Treatment with non-targeting gRNAs did not result in detectable indels in the SMAD3 genomic region, thereby demonstrating the specificity of the system on this target. Despite well-known variability in TD efficiencies between different cell types, the relative difference in indel frequency achieved with the three tested SMAD3-specific gRNAs was comparable between HBECs and NHLFs, potentially suggesting a broad applicability of the approach.

**Fig 2:**
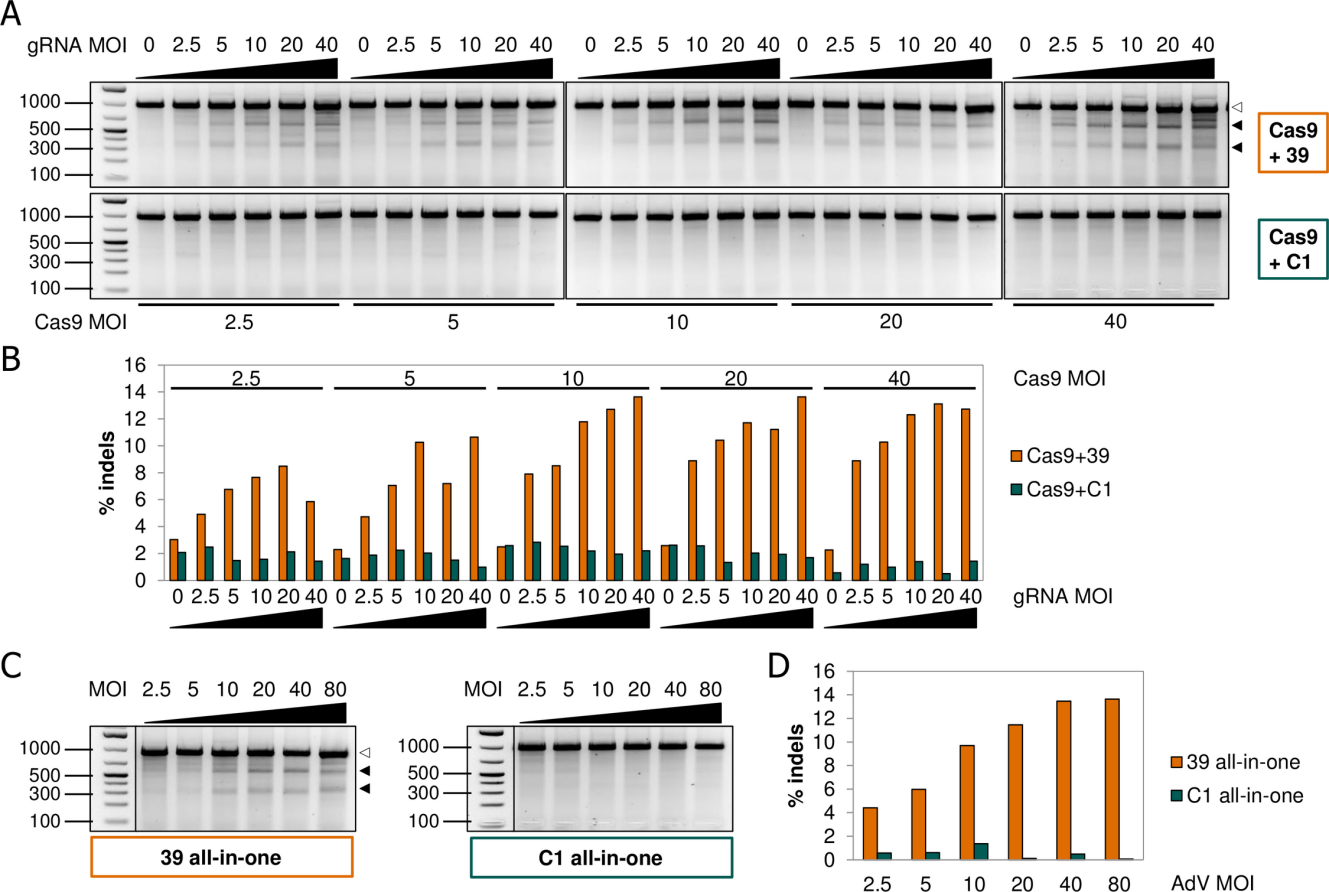
Titration of Cas9 and gRNA AdV effects on SMAD3 indel formation in NHLFs. (**A**) Primary NHLFs were co-transduced at DIV 1 with varying ratios of Cas9 and gRNA AdVs at a maximal total MOI of 80. NHLFs were harvested at DIV 9 after which genomic DNA was used in PCR amplification of the SMAD3 target region. Resulting PCR products were used for SURVEYOR^®^ assay analysis and resolved by agarose gel electrophoresis. A single full-length band at 933 bp (highlighted with white arrowheads) indicates the uncleaved PCR product, whereas appearance of two additional bands following treatment with SURVEYOR^®^ nuclease indicates the presence of indels (highlighted with black arrowheads). (**B**) Quantification of indel frequencies as determined by densitometry of the DNA fragments depicted in panel A. (**C**) NHLFs were treated as in A, except that TD was performed with a single all-in-one AdV particle. (D) Quantification of indel frequencies as determined by densitometry of the DNA fragments depicted in panel C. 39: gRNA targeting SMAD3; C1: gRNA targeting control sequences.

To study the effects of SMAD3 genomic editing on functional readouts in human primary cells, we first needed to determine the optimal conditions for editing. To that end, NHLFs were co-transduced with Cas9 and either SMAD3_v39 or non-targeting gRNA AdVs at various MOIs ranging from 2.5–40 for each viral particle. In parallel, NHLFs were transduced with a single all-in-one AdV containing Cas9 plus SMAD3_v39 or non-targeting gRNA at MOIs ranging from 2.5–80. Following TD with the appropriate AdV construct(s) SMAD3 genomic editing was analyzed with the use of the SURVEYOR^®^ assay as described earlier. In general, up to the mid-MOI range tested the use of a higher MOI for TD of the NHLFs increased indel formation in the SMAD3 target region (Fig 2A–2D). This was true for both the co-TD and all-in-one approach. For the comparison of co-TD with the all-in-one AdV, it needs to be taken into account that equal molarities of AdVs will result in higher molar ratios of Cas9 and gRNA, due to the presence of both inserts in one particle. Thus co-TD MOIs of 5 + 5 would carry molar equivalents of all-in-one AdV MOI 5 for both inserts. Interestingly, these two approaches yielded similar levels of indel formation at equimolar levels of insert (S9A Fig). Apart from genomic editing, toxicity was assessed by performing a cell viability assay to determine the AdV MOI which could be relevant for use in functional assays. Delivery of AdV constructs in NHLFs by co-TD or single TD resulted in similar amounts of cellular toxicity which was limited to about 20% for the highest viral load tested (S4 Fig). The TD efficiency in NHLFs was close to 100% already at MOI 10, according to titrations using an AsRed reporter-gene AdV (S6A Fig). Therefore, the optimal MOI in NHLFs for Cas9 and gRNA AdVs is expected to be in the range of MOI 10–20 each for functional studies.

**Fig 3:**
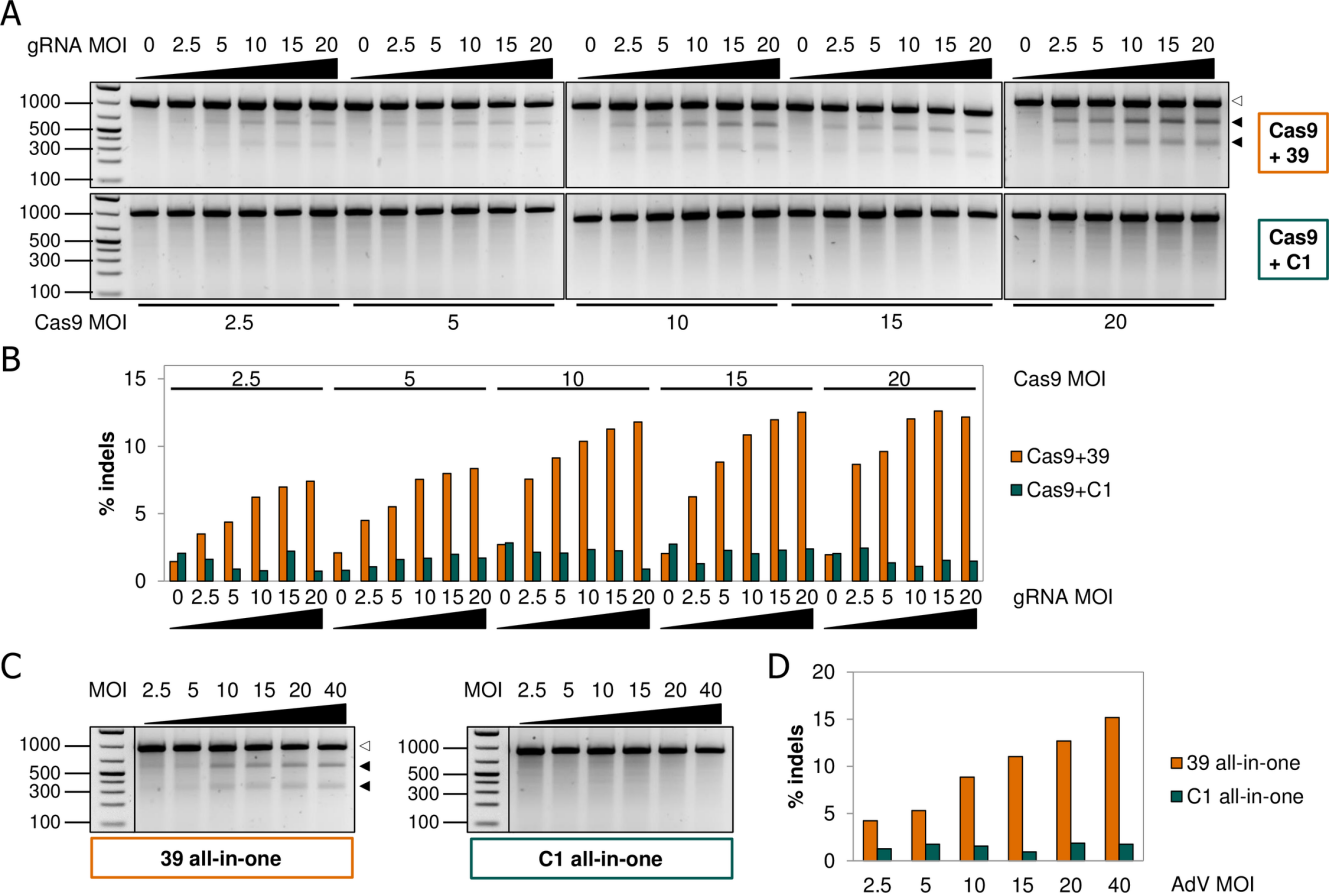
Titration of Cas9 and gRNA AdV effects on SMAD3 indel formation in HBECs. (**A**) Primary HBECs were co-transduced at DIV 1 with varying ratios of Cas9 and gRNA AdV at a maximal total MOI of 40. HBECs were harvested at DIV 9 after which genomic DNA was analyzed for the presence of indels by SURVEYOR^®^ assay as described earlier. (**B**) Quantification of indel frequencies as determined by densitometry of the DNA fragments depicted in panel A. (**C**) HBECs were treated as in A, except that TD was performed with a single all-in-one AdV particle. (D) Quantification of indel frequencies as determined by densitometry of the DNA fragments depicted in panel C. White arrowheads indicate the positions of parental PCR fragments whereas black arrowheads mark the SURVEYOR^®^ nuclease digested fragments containing indels. 39: gRNA targeting SMAD3; C1: gRNA targeting control sequences.

Since the optimal condition may be different for each cell type, a similar AdV dose-response curve was generated for the HBECs as well. Due to the higher toxicity in these cells, the viral load range was lowered to MOI 2.5–20 for the co-TD and MOI 2.5–40 for TD with the all-in-one AdV constructs (S5 Fig). Like in NHLF cells, NHEJ-derived indel formation in the SMAD3 gene was increased with higher viral load used for delivery of Cas9 and gRNA (Fig 3A–3D). A plateau was reached in the mid-MOI range for the co-TD approach, but was less evident for the all-in-one system. Generally speaking, co-TD of HBECs with Cas9 and SMAD3_v39 gRNA AdVs or TD with all-in-one SMAD3_v39 AdV resulted in comparable genomic editing levels at equimolar levels of insert (S9B Fig). The all-in-one system may, however, perform somewhat better than the co-TD approach especially at the lower MOI range. This advantage may be offset by slightly higher toxicity of the all-in-one system versus co-TD though (see S4 and S5 Figs). Remarkably, the overall efficiency of indel formation in HBECs at lower total MOI compared to NHLFs was similar, although TD-efficiencies appeared significantly lower for HBECs when tested with AsRed reporter AdV (S6B Fig). Taking these data into account, we predict the optimal MOI for Cas9 and gRNA AdVs in HBECs to be lower than that used for the NHLFs to enable functional readouts. To summarize, these data demonstrate that AdV delivery of CRISPR/Cas9 components results in very efficient gene interference in human primary cells, without the need for selection or enrichment of edited cells.

### AdV-delivered CRISPR/Cas9 components result in effective depletion of SMAD3 protein in human primary cells

**Fig 4:**
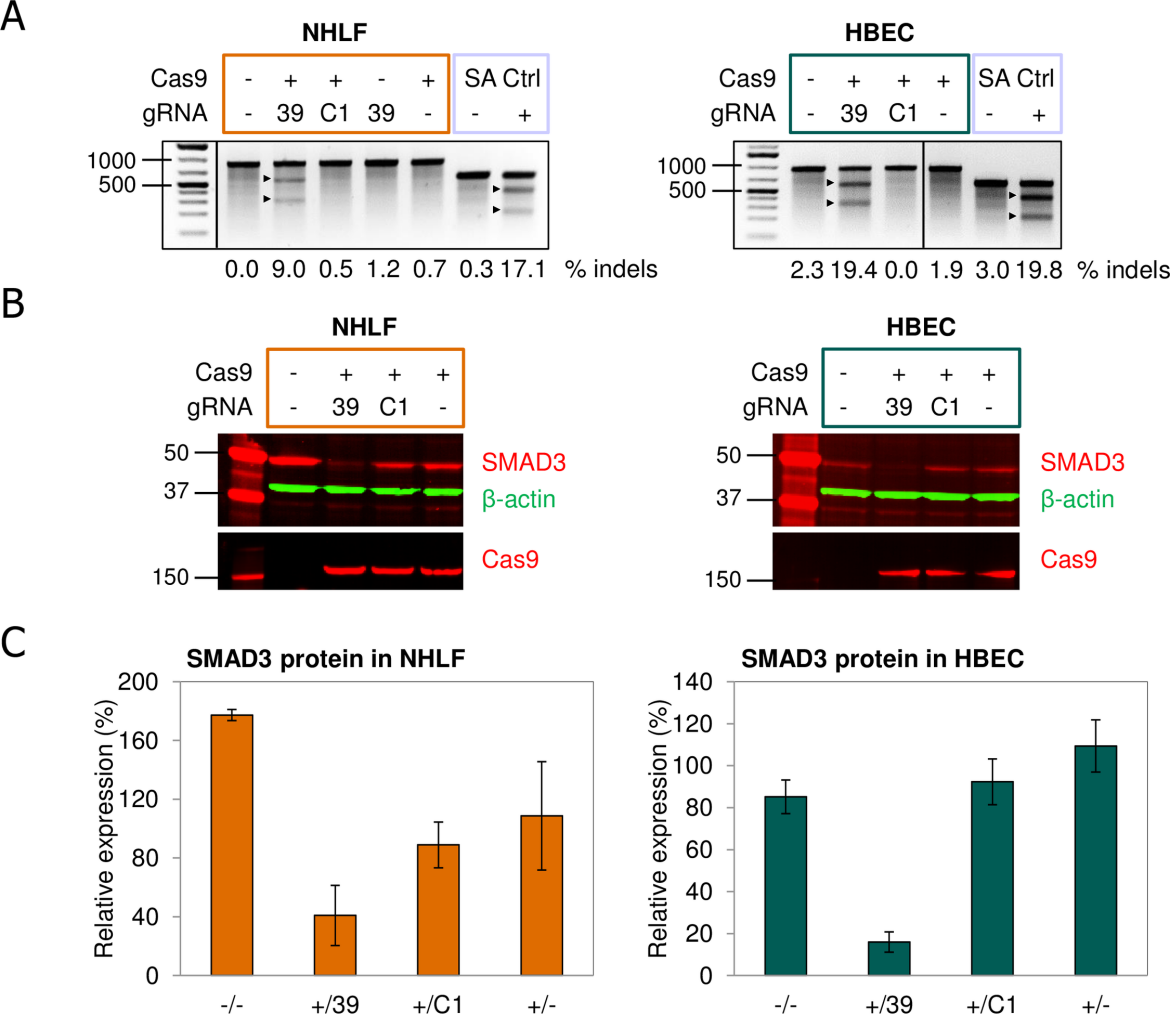
CRISPR/Cas9-mediated depletion of SMAD3 protein in primary NHLFs and HBECs. (**A**) SMAD3 genomic editing in NHLFs and HBECs following co-TD with Cas9 and SMAD3_v39 gRNA AdV. Primary NHLFs (left) and HBECs (right) were co-transduced with Cas9 and either SMAD3 or control gRNA AdV at total MOI 30 (Cas9:gRNA ratio 1:2) after which genomic DNA was analyzed for the presence of indels at DIV 12 by SURVEYOR^®^ assay as described earlier. Indel frequencies are shown below each lane and were determined by densitometry of the full-length and cleaved PCR fragments. 39: gRNA targeting SMAD3; C1: gRNA targeting control sequence; SA Ctrl: SURVEYOR® assay control. (**B**) AdV CRISPR/Cas9-mediated SMAD3 protein KD in NHLFs and HBECs. Cells were harvested at DIV 12 for protein analysis by immunoblotting. A total of 35 μg or 28 μg protein per lane was loaded for the NHLFs and HBECs, respectively. Immunoblots were stained with the indicated primary antibodies. (**C**) Quantification of SMAD3 protein levels in NHLFs and HBECs treated with Cas9 and SMAD3-targeting AdV. SMAD3 protein expression was quantified by densitometry of the bands shown in panel B. Expression is blotted relative to the control (co-TD with Cas9 and control gRNA C1). Data points are from biological duplicates (n = 2) and error bars represent standard deviation.

DNA double strand breaks generated by the wild type Cas9 protein complexed with non-coding gRNA are repaired by the error-prone NHEJ pathway in most cases. This will lead to indels in the target region, which may or may not interfere with expression of the target gene. In general, indels generated by the NHEJ repair pathway should result in a frameshift in the open reading frame in two-thirds of the cases. Very often this will lead to KO of full-length protein expression, as a premature stop codon is generated, causing either expression of a truncated, non-functional protein or degradation of the mRNA by the nonsense-mediated decay pathway [26]. Although high frequencies of indel formation were obtained in the targeted SMAD3 genomic region using AdV delivery of CRISPR/Cas9 components, this may not necessarily be reflected on the protein level, which is most relevant for phenotypic assays. Therefore, the protein expression level of SMAD3 in NHLFs and HBECs was assessed following co-TD with Cas9 and one of the SMAD3-specific gRNAs (v39), which was most effective in generating indels. On DIV 12 after co-TD, cells were harvested for genomic DNA analysis as well as protein analysis. As seen previously, efficient disruption of the targeted SMAD3 gene was observed, given the level of indel formation in both cell types (Fig 4A). Analysis of protein lysates from these cells by immunoblotting revealed highly efficient depletion of SMAD3 protein after co-TD with Cas9 and SMAD3-targeting gRNA (Fig 4B). In contrast, TD of the cells with Cas9 alone or in combination with control gRNA AdV did not lead to a specific reduction in SMAD3 protein levels correlated with indel formation, although SMAD3 protein levels appear to be non-specifically reduced by AdV TD in NHLFs, but not HBECs. This does not seem to be related to Cas9 expression, but is a general effect caused by AdV TD (S7 Fig). The specific effect on SMAD3 protein expression is reproducible in both cell types and has been observed in two independent experiments. Detection of Cas9 and β-actin protein as loading control on the immunoblots suggests equal loading of samples and strong presence of Cas9 when expressed. The efficient depletion of SMAD3 protein following AdV co-TD of Cas9 and SMAD3-specific gRNA is reflected in quantification of the SMAD3 signals by densitometry (Fig 4C). The effect was most noticeable in HBECs, where almost 90% reduction was seen. Although less pronounced in NHLF cells, about 60% reduction in SMAD3 expression is observed compared to control gRNA. This suggests that AdV delivery of CRISPR/Cas9 components in human primary cells can result in highly efficient and fast targeted depletion of protein expression without enrichment of edited cells.

### The AdV CRISPR/Cas9 platform is a valuable tool for application in phenotypic assays

**Fig 5:**
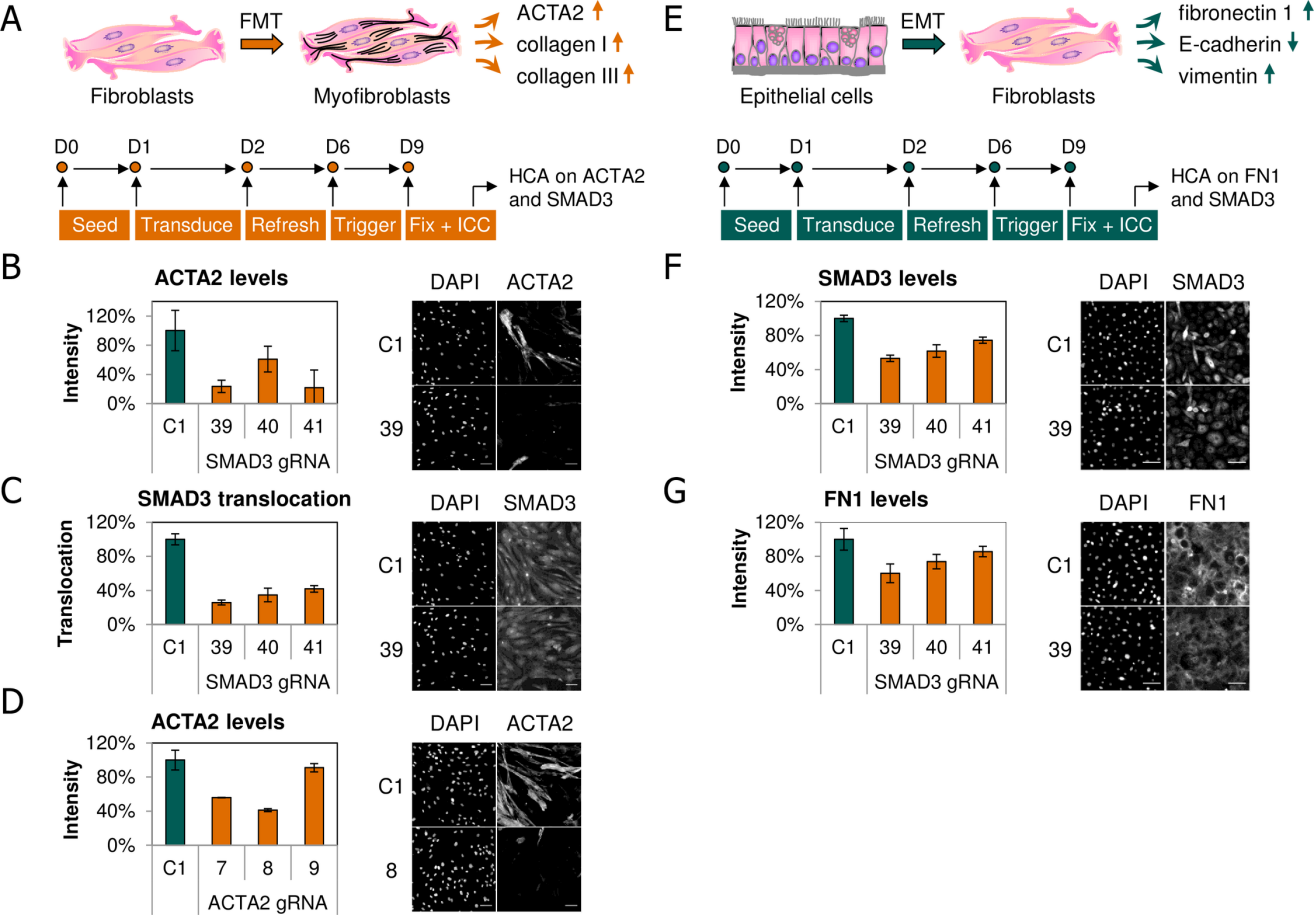
Application of CRISPR/Cas9 to human primary cell phenotypic assays. (**A**) Overview of the FMT assay with indicated time points of treatment in days. Upregulation of markers specific for myofibroblasts is indicated with upward arrows. At the end of the assay HCA is performed on ACTA2 and SMAD3. (**B-D**) FMT phenotypic assay with HCA on ACTA2 and SMAD3. Primary NHLFs were co-transduced at DIV 1 with Cas9 and either SMAD3, ACTA2 or control gRNA AdV at total MOI 30 (Cas9:gRNA ratio 1:2), followed by addition of TGF-β1 at DIV 6. Cells were fixed for immunofluorescent labelling of SMAD3 and ACTA2 followed by HCA at DIV 9. Graphs are representing HCA quantification of ACTA2 and SMAD3 immunofluorescent intensities in optimized segmentation masks and are blotted relative to the control (C1). Data points are from triplicate wells (n = 3) and error bars represent standard deviation. Exemplifying images of target protein and nuclear DAPI labelling are shown. (**E**) Overview of the EMT assay with indicated time points of treatment in days. Change in expression of certain markers is shown, with arrows demonstrating up- or downregulation (upward and downward arrows, respectively). At the end of the assay HCA is performed on FN1 and SMAD3. (**F-G**) EMT phenotypic assay with HCA on FN1 and SMAD3. Primary HBECs were co-transduced at DIV 1 with Cas9 and either SMAD3 or control gRNA AdV at total MOI 12 (Cas9:gRNA ratio 1:1), followed by addition of a cocktail containing TGF-β1 and TNFα at DIV 6. Cells were fixed and stained as described in panel A, except that staining was performed for FN1 and SMAD3 in this case. Graphs are blotted relative to the control (C1). Scale bars indicate 100 μm. 39, 40, 41: gRNAs targeting SMAD3; 7, 8, 9: gRNAs targeting ACTA2; C1: gRNA targeting control sequence.

To explore its use for target identification and/or validation, the AdV CRISPR/Cas9 platform was applied to phenotypic assays, which mimic some molecular aspects observed in fibrosis. In the FMT assay, NHLFs were treated with the pro-fibrotic stimulus TGF-β1, resulting in differentiation of the cells into myofibroblasts which are characterized by expression of ACTA2, secretion of extracellular matrix (ECM) components, such as collagen I and III, and increased migration and contractility (Fig 5A) [27]. Being one of the key players in the TGF-β signaling pathway, SMAD3 is involved in inducing the expression of ACTA2 and ECM components [28,29]. Prior to TGF-β1 stimulation on DIV 6 NHLFs were co-transduced with Cas9 and different gRNA AdV constructs on DIV 1, and ACTA2 and SMAD3 were monitored by HCA at the end of the assay. The MOI used was based on the results shown in Fig 2. Treatment of NHLFs with TGF-β1 alone led to a significant increase in the number of myofibroblasts which is evident from the appearance of ACTA2 expression (S8A Fig). This increase in ACTA2 was reduced to almost background level by SMAD3-specific gRNAs, but not the control gRNA (Fig 5B). All three tested SMAD3 gRNAs had a suppressive effect on ACTA2 expression with SMAD3_v39 being the most potent, in agreement with the effect on indel formation (S1A Fig). Similar results were obtained in two independent experiments. These results suggest that CRISPR/Cas9-mediated SMAD3 KD can inhibit fibroblast to myofibroblast transition in the FMT assay. In addition, SMAD3 nuclear translocation was significantly reduced by SMAD3-specific gRNA, but not the control gRNA (Fig 5C and S2 Fig). Since nuclear translocation of SMAD3 is required for regulating expression of TGF-β1 target genes, this provides an explanation for the effects seen on ACTA2 expression. As a positive control we also treated cells with Cas9 and gRNAs targeting ACTA2. Two of three tested ACTA2 gRNAs effectively decreased expression of ACTA2 protein in line with indel formation (Fig 5D and S1A Fig), supporting the relevance of the approach.

Apart from the FMT assay we also applied the AdV CRISPR/Cas9 platform in an EMT assay, using primary HBECs. The EMT assay is based on the transition of epithelial to mesenchymal cells, which is primarily induced by activation of the TGF-β pathway. During this process epithelial cells lose their epithelial phenotype and acquire fibroblast-like properties, as well as increased motility and reduced cell adhesion. Epithelial cells undergoing EMT display decreased expression of epithelial markers (e.g. E-cadherin and ZO-1) and increased expression of mesenchymal markers, such as collagen I and III and FN1 (Fig 5E) [27]. For EMT induction the experimental setup was similar to the FMT assay, except that the cells were stimulated with a cocktail of TGF-β1 and TNFα (tumor necrosis factor alpha), instead of TGF-β1 alone. Treatment of HBECs with TGF-β1 and TNFα together led to an increase in the expression of FN1, a well-established mesenchymal marker downstream of SMAD3 (S8B Fig). Co-TD of HBECs with Cas9 and SMAD3-targeting gRNA led to strong depletion of SMAD3 protein with SMAD3_v39 again being the most potent gRNA (Figs 5F and S1B). The MOI used was based on the results shown in Fig 3. Parallel observations were made for FN1, where the different SMAD3-specific gRNAs showed a similar trend in reducing FN1 expression (Fig 5G) [27]. Comparable results were obtained in two independent experiments. These data indicate a significant inhibition of EMT in HBECs treated with our AdV CRISPR/Cas9 constructs through inactivation of SMAD3 and, as a result, the TGF-β pathway. Thus, AdV delivery of the CRISPR/Cas9 system in human primary cells appears to be efficient and may be a valuable tool for target discovery and/or target validation purposes, which may contribute substantially to the drug discovery process.

## Discussion

Drug discovery is in part dependent on the identification and validation of novel targets, which until now has largely relied on the RNAi technology, using siRNA or shRNA to target the mRNA of interest. Since its discovery in the late 1990s, application of this technology in disease-relevant phenotypic assays has led to the identification of many interesting and valid targets in several therapeutic areas. Although RNAi has been shown to be a powerful method, its limitations have also increasingly surfaced. RNAi acts at the post-transcriptional level targeting the mRNA, whereas CRISPR/Cas9 exerts its effects upstream at the genomic DNA level. As a consequence, CRISPR/Cas9 effects are heritable in dividing cells and may result in amplification of the initial impact, thereby obtaining potentially greater efficiencies when compared to RNAi. While RNAi may have different effects on the transcript variants of its target, CRISPR/Cas9 can lead to KD of all mRNA isoforms from the same gene. Since CRISPR/Cas9 is mechanistically different from RNAi it may also have other unwanted off-target effects. In many cases these off-targets may not be relevant since the majority of the genome is not expressed. Combining the results obtained by using CRISPR/Cas9 with those obtained by RNAi may also assist in identifying specific effects of target KD/KO.

Since the recent first publications on the mechanism of DNA cleavage by the CRISPR/Cas9 system, the application of this RGEN has reached unprecedented levels [8,30,31]. The technology is widely applicable, which is illustrated by multiple studies reporting on the generation of genomic editing and KOs in cells, but also in different animal species, ranging from *Caenorhabditis elegans* and *Drosophila* to mouse and primates [32–37], as well as plants. The main advantage of the CRISPR/Cas9 system compared to other nucleases is the simplicity and low cost of its application. For every different target of interest only the 19–20 nucleotide target specific sequence of the gRNA has to be adjusted, making the CRISPR/Cas9 platform a particularly attractive system for high-throughput purposes. This is different from ZFNs or TALENs, where completely new proteins have to be designed for each different target site.

As CRISPR/Cas9 is present in prokaryotes only, its application in eukaryotes requires introduction of both Cas9 and gRNA into the host. Delivery of these components can be achieved by different means, such as plasmid transfection or viral TD. The latter method clearly has the most potential due to its use in many cell types, including dividing and non-dividing cells, its relatively high delivery efficiency and its application in high-throughput screening. Successful delivery of Cas9 and gRNA by different viral vectors has been reported, such as LVs [10,11], adeno-associated virus (AAV) [38] and AdV [39]. However, the AdV delivery method seems to outperform the accuracy of the other available systems when it comes to HDR to establish gene editing [12]. The implementation of CRISPR/Cas9 in human differentiated primary cells has so far been limited to only a few different fully differentiated cell types, including CD4+ T-lymphocytes, dermal fibroblasts, (pre)adipocytes, endothelial cells, and airway epithelial cells [15–19]. In many of these cases, selection or sorting of edited cells was applied to increase efficiency [19,40]. Such selection procedures are not always desirable, since they may affect the properties of the primary cells and are time consuming. As target discovery and drug evaluation in such types of cells appears to be most relevant, we were interested to determine whether AdV delivery of the CRISPR/Cas9 system without selection of manipulated cells is of sufficient efficiency to study phenotypic changes in human primary cells. Taking this into account, we have set up an AdV-based CRISPR/Cas9 system to enable target identification and validation for drug discovery.

In this study we demonstrate the power of this system as a proof of principle by focusing on SMAD3, a well-known player in the TGF-β signaling pathway. Our results show highly efficient genomic editing of SMAD3 at the DNA level in two different human primary cell types without enrichment or selection of the edited cell population. Genomic editing increased at higher viral load, but reached a plateau around a total MOI 40 and 20 in the NHLFs and HBECs, respectively. Under these conditions most of the cells should be transduced with AdV and are therefore expected to express Cas9 and gRNA. One advantage of the co-TD system is that it allows varying the ratio between Cas9 and gRNA expression within the cell. This is different from the all-in-one AdV where Cas9 and gRNA ratios are fixed. Direct comparison of TD with a single all-in-one Cas9/gRNA AdV versus co-TD with separate Cas9 and gRNA AdVs revealed, that both strategies work equally well when it comes to genomic editing. These results suggest that the genomic disruption is mainly dependent on the overall concentration of the Cas9-gRNA complex rather than a specific ratio of the endonuclease and gRNA components, which is in line with other reports [39]. The use of a single all-in-one AdV may, however, elicit higher toxicity (S4 and S5 Figs), potentially due to technical aspects of AdV production, and will have to be further improved, but may offer significant advantages over co-TD approaches in the future, in particular for high-throughput screening. Genetic disruption of SMAD3 using the AdV-based CRISPR/Cas9 system led to strongly reduced protein expression, suggesting that the majority of NHEJ-derived indels resulted in protein KO. The depletion of SMAD3 protein inhibited downstream events in the TGF-β signaling cascade, where ACTA2 and FN1 levels were significantly decreased in NHLFs and HBECs, respectively. These results indicate suppression of SMAD3 expression by AdV CRISPR/Cas9 leading to the desired phenotype, i.e. inhibition of FMT/EMT, both of which are processes contributing to the development of fibrosis *in vivo*. Taken together, the AdV CRISPR/Cas9 platform can be considered an elegant way to study the contribution of genes to the development of a certain disease state and may, therefore, expand the current target discovery and validation toolbox.

As with RNAi, the CRISPR/Cas9 system is expected to have off-target effects, since the guide sequence is only 19–20 nucleotides in length. Reports concerning Cas9 off-targeting have been somewhat contradicting so far. While some studies mention significant off-target mutagenesis, others demonstrate relatively low indel frequencies at unintended genomic regions [41–43]. It appears that binding of Cas9 to a certain site does not necessarily result in cleavage and, therefore, generation of a DSB. Although binding of Cas9 can be widespread (i.e. varying from 10 to >1000 sites), the vast majority of off-target sites do not appear to be mutated [44,45]. This is an important finding, since the incidence of off-target mutations should be minimized if one aims at studying gene function or, more importantly, wishes to correct a disease-causing mutation. In the current study we have not investigated off-target effects, which will have to be carried out in future studies to fully evaluate the system.

The effects seen after CRISPR/Cas9-mediated editing of SMAD3 appeared robust in the FMT phenotypic assay, whereas the outcome was less pronounced in the EMT assay. The latter may be due to the relatively low MOI used for TD of the cells. The HBECs were treated with MOI 12 in the actual EMT phenotypic assay to limit unspecific effects in the complex assay and to keep the cells viable (Figs 5F and 5G, S1B and S5A). The lower MOI used in the EMT assay may have yielded less protein KD. As a consequence, fibronectin 1 expression is only moderately affected with about 40% reduction at most in case of treatment with SMAD3_v39 gRNA. Future optimization of the assay will have to address these issues, which could include the use of an all-in-one AdV or the use of purified rather than crude AdV. The FMT phenotypic data appear to show a correlation with the observed SMAD3 indel levels and the resulting effect on protein abundance. In general the SMAD3_v39 gRNA consistently yielded the highest efficiency, both in HBEC and NHLF primary cells. Since the targeted gene disruption leads to prominent downstream inhibition of ACTA2 expression in the FMT assay, it can be concluded that AdV CRISPR/Cas9 efficiently inactivates SMAD3. It should be emphasized again that this effect was achieved without selection or enrichment of Cas9 and/or gRNA expressing or edited cells, which is often performed to increase gene editing efficiency. Therefore, the AdV CRISPR/Cas9 system is expected to offer significant opportunities beside of the current available strategies in drug discovery. The combined use of RNAi and CRISPR/Cas9 for target identification and validation may boost the drug discovery process further and open entire new opportunities. It will be interesting to see what this strategy will bring in the future.

## Supporting information

S1 Fig Indel analysis of FMT and EMT derived material. (**A**) Genomic editing of the SMAD3 and ACTA2 genes in NHLFs co-transduced with Cas9 and SMAD3- or ACTA2-targeting gRNA AdV. Cells were co-transduced at DIV 1 at total MOI 30 (Cas9:gRNA ratio 1:2), followed by addition of TGF-β1 at DIV 6. Genomic DNA was analyzed for the presence of indels at DIV 9 by SURVEYOR^®^ assay as described earlier. Black arrowheads mark the DNA fragments that appear after successful gene editing. Indel frequencies are shown below each lane and were determined by densitometry of the full-length and cleaved PCR fragments as described above. (**B**) Genomic editing of the SMAD3 gene in HBECs co-transduced with Cas9 and SMAD3-targeting gRNA AdV. Cells were co-transduced at total MOI 12 (Cas9:gRNA ratio 1:1) followed by EMT induced by addition of a cocktail containing TGF-β1 and TNFα at DIV 6. Genomic DNA was analyzed for the presence of indels at DIV 9 by SURVEYOR^®^ assay as described earlier. 39, 40, 41: gRNAs targeting SMAD3; 7, 8, 9: gRNAs targeting ACTA2; C1: gRNA targeting control sequence; SA Ctrl: SURVEYOR^®^ assay control.
(TIFF)

S2 Fig Inhibition of SMAD3 nuclear translocation. Visualization of SMAD3 nuclear translocation in NHLFs co-transduced with Cas9 and SMAD3-targeting gRNA AdV. Primary NHLFs were co-transduced at DIV 1 at total MOI 30 (Cas9:gRNA ratio 1:2), followed by addition of TGF-β1 at DIV 6. Cells were fixed for immunofluorescent labelling of SMAD3 and nuclear DAPI staining. Exemplifying images of SMAD3 and nuclear DAPI labelling are shown. Translocated SMAD3 is highlighted with orange arrowheads. 39: gRNA targeting SMAD3; C1: gRNA targeting control sequence.
(TIF)

S3 Fig CRISPR/Cas9 AdV-mediated gene disruption in two human primary cell types. (**A**) Genomic editing of the SMAD3 gene in NHLFs following co-TD with Cas9 and SMAD3-targeting gRNA AdV. NHLFs were co-transduced at DIV 1 at total MOI 30 (Cas9:gRNA ratio 1:2) followed by genomic DNA isolation at DIV 7 and PCR amplification of the SMAD3 target region. Resulting PCR products were used for SURVEYOR^®^ assay analysis and resolved by agarose gel electrophoresis as described earlier. Indel frequencies are shown below each lane determined by densitometry of the full-length and cleaved PCR fragments (black arrowheads). (**B**) Genomic editing of the SMAD3 gene in HBECs co-transduced with Cas9 and SMAD3-targeting gRNA AdV. Primary HBECs were co-transduced as above, but with a total MOI 14 (Cas9:gRNA ratio 1:2.5). Genomic DNA was analyzed for the presence of indels at DIV 4 by SURVEYOR^®^ assay as described above. 39, 40, 41: gRNAs targeting SMAD3; C1, C2: gRNAs targeting control sequences; SA Ctrl: SURVEYOR^®^ assay control.
(TIFF)

S4 Fig AdV TD-mediated toxicity in NHLFs. Assessment of toxicity resulting from TD of NHLFs with varying amounts of AdV. (**A**) Primary NHLFs were co-transduced at DIV 1 with varying ratios of Cas9 and gRNA AdV or AsRed and gRNA AdV at a maximal total MOI of 80 followed by addition of TGF-β1 at DIV 6. Cell viability was determined at DIV 9 by using the CellTiter-Blue^®^ Cell Viability Assay. Data are normalized to the untreated condition. (**B**) NHLFs were treated as in A, except that cells were either co-transduced or transduced with the single all-in-one AdV. For the co-TD approach only the data from Cas9:gRNA AdV ratio of 1:1 are utilized for comparison with the all-in-one system. (**C**) NHLFs were treated as in A, except that cells were transduced with a single all-in-one Cas9/gRNA AdV or an AdV particle without insert (“empty”). For the comparison of co-TD (ratio 1:1) with the all-in-one approach, it is important to note that equal MOIs will result in double molar amounts of Cas9 and gRNA with the all-in-one AdV, as the total MOI is kept constant. Data points are from quadruplicate wells (n = 4) and error bars represent standard deviation.
(TIFF)

S5 Fig AdV TD-mediated toxicity in HBECs. Assessment of toxicity resulting from TD of HBECs with varying amounts of AdV. (**A**) Primary HBECs were co-transduced at DIV 1 with varying ratios of Cas9 and gRNA AdV or AsRed and gRNA AdV at a maximal total MOI of 40 followed by addition of a cocktail containing TGF-β1 and TNFα at DIV 6. Cell viability was determined at DIV 9 by using the CellTiter-Blue^®^ Cell Viability Assay. Data are normalized to the untreated condition. (**B**) HBECs were treated as in A, except that cells were either co-transduced or transduced with the single all-in-one AdV. For the co-TD approach only the data from Cas9:gRNA AdV ratio of 1:1 are utilized for comparison with the all-in-one system. (**C**) HBECs were treated as in A, except that cells were transduced with a single all-in-one Cas9/gRNA AdV or an AdV particle without insert (“empty”). For the comparison of co-TD (ratio 1:1) with the all-in-one approach, it is important to note that equal MOIs will result in double molar amounts of Cas9 and gRNA with the all-in-one AdV, as the total MOI is kept constant. Data points are from quadruplicate wells (n = 4) and error bars represent standard deviation.
(TIFF)

S6 Fig Dose-response and TD efficiency in NHLFs and HBECs. Determination of TD efficiencies using an AsRed reporter-gene AdV. (**A**) NHLFs were transduced at DIV 1 with an AsRed AdV at various MOIs ranging from 2.5–40, after which cells were fixed and stained with DAPI at DIV 9. Cells were imaged to determine the number of viable, AsRed-positive cells by HCA (see Materials and Methods for details). (**B**) HBECs were transduced at DIV 1 with an AsRed AdV at various MOIs ranging from 2.5–20 at DIV 1, after which cells were fixed and stained with DAPI at DIV 9. Cells were imaged to determine the number of viable, AsRed-positive cells by HCA as above (see Materials and Methods for details). Data points are from six biological replicates (n = 6 wells) and error bars represent standard deviation.
(TIFF)

S7 Fig Specificity of SMAD3 downregulation by AdV TD in NHLFs. (**A**) SMAD3 genomic editing in NHLFs following co-TD with Cas9 or AsRed and gRNA AdV or single TD with an “empty” AdV particle (no insert). Primary NHLFs were transduced at total MOI 30 (Cas9:gRNA ratio 1:2) after which genomic DNA was analyzed for the presence of indels at DIV 8 by SURVEYOR^®^ assay as described earlier. (**B**) AdV CRISPR/Cas9-mediated SMAD3 protein KD in NHLFs. Cells were harvested at DIV 8 for protein analysis by immunoblotting. A total of 30–35 μg protein per lane was loaded. Immunoblots were stained with the indicated primary antibodies. (**C**) Quantification of SMAD3 protein levels by densitometry of the bands depicted in panel B. Expression is blotted relative to the untreated condition and normalized to the β-actin signal. Data points are from biological duplicate samples (n = 2) and error bars represent standard deviation.
(TIFF)

S8 Fig Induction of FMT and EMT. (**A**) NHLFs were triggered at DIV 6 with TGF-β1 to induce FMT and fixed at DIV 9 followed by immunofluorescent labelling of ACTA2 and DAPI nuclear staining. Representative images of the triggered (T+) and untriggered (T-) conditions are shown. (**B**) HBECs were triggered at DIV 6 with a cocktail containing TGF-β1 and TNFα to induce EMT and fixed at DIV 9 followed by immunofluorescent labelling of FN1 and DAPI nuclear staining. Representative images of the triggered (T+) and untriggered (T-) conditions are shown. Scale bars indicate 100 μm.
(TIFF)

S9 Fig Comparison of SMAD3 indel formation achieved with co-TD and all-in-one AdV approach in NHLFs and HBECs. (**A**) Primary NHLFs were either co-transduced or transduced with an all-in-one AdV at DIV 1 at a maximal total MOI of 80. In case cells were co-transduced with Cas9 and gRNA AdV the ratio of Cas9:gRNA was 1:1 to allow for direct comparison with the all-in-one AdV. NHLFs were harvested at DIV 9 after which genomic DNA was analyzed for the presence of indels by SURVEYOR^®^ assay as described earlier. Indel frequencies were quantified as determined by densitometry of the DNA fragments depicted in Fig 2A and 2C. (**B**) Primary HBECs were treated as in A, except that TD was performed at a maximal total MOI of 40. Indel frequencies were quantified as determined by densitometry of the DNA fragments depicted in Fig 3A and 3C. Cas9+39: co-TD with Cas9 and SMAD3 targeting gRNA; 39 all-in-one: TD with a SMAD3 targeting all-in-one AdV.
(TIFF)

S1 Table Primer and gRNA targeting sequences used in this study. The expected PCR fragments after successful genomic editing detected by SURVEYOR^®^ assay are shown. All gRNAs are preceded by a guanine nucleotide (underlined) at the 5’ end to promote transcription driven by the U6 promoter. NA: not applicable.
(DOCX)

## References

[1] Es H van, Arts G. Biology calls the targets: combining RNAi and disease biology. Drug Discov Today. 2005;10: 1385–1391. doi: 10.1016/S1359-6446(05)03590-7 16253877 10.1016/S1359-6446(05)03590-7

[2] Berns K, Hijmans EM, Mullenders J, Brummelkamp TR, Velds A, Heimerikx M, et al A large-scale RNAi screen in human cells identifies new components of the p53 pathway. Nature. 2004;428: 431–437. doi: 10.1038/nature02371 15042092 10.1038/nature02371

[3] Jackson AL, Bartz SR, Schelter J, Kobayashi S V, Burchard J, Mao M, et al Expression profiling reveals off-target gene regulation by RNAi. Nat Biotechnol. 2003;21: 635–637. doi: 10.1038/nbt831 12754523 10.1038/nbt831

[4] Ohneda K, Moriguchi T, Ohmori S, Ishijima Y, Satoh H, Philipsen S, et al Transcription factor GATA1 is dispensable for mast cell differentiation in adult mice. Mol Cell Biol. 2014;34: 1812–26. doi: 10.1128/MCB.01524-13 24615013 10.1128/MCB.01524-13 PMC4019035

[5] Gaj T, Gersbach C a., Barbas CF. ZFN, TALEN, and CRISPR/Cas-based methods for genome engineering. Trends Biotechnol. Elsevier Ltd; 2013;31: 397–405. doi: 10.1016/j.tibtech.2013.04.004 23664777 10.1016/j.tibtech.2013.04.004 PMC3694601

[6] Sander JD, Joung JK. CRISPR-Cas systems for editing, regulating and targeting genomes. Nat Biotechnol. 2014;32: 347–55. doi: 10.1038/nbt.2842 24584096 10.1038/nbt.2842 PMC4022601

[7] Barrangou R, Fremaux C, Deveau H, Richards M, Boyaval P, Moineau S, et al CRISPR provides acquired resistance against viruses in prokaryotes. Science. 2007;315: 1709–1712. doi: 10.1126/science.1138140 17379808 10.1126/science.1138140

[8] Jinek M, Chylinski K, Fonfara I, Hauer M, Doudna J a., Charpentier E. A Programmable Dual-RNA-Guided DNA Endonuclease in Adaptive Bacterial Immunity. Science (80-). 2012;337: 816–821. doi: 10.1126/science.1225829 22745249 10.1126/science.1225829 PMC6286148

[9] Mojica FJM, Díez-Villaseñor C, García-Martínez J, Almendros C. Short motif sequences determine the targets of the prokaryotic CRISPR defence system. Microbiology. 2009;155: 733–740. doi: 10.1099/mic.0.023960-0 19246744 10.1099/mic.0.023960-0

[10] Shalem O, Sanjana NE, Hartenian E, Shi X, Scott D a, Mikkelsen TS, et al Genome-scale CRISPR-Cas9 knockout screening in human cells. Science. 2014;343: 84–7. doi: 10.1126/science.1247005 24336571 10.1126/science.1247005 PMC4089965

[11] Wang T, Wei JJ, Sabatini DM, Lander ES. Genetic screens in human cells using the CRISPR-Cas9 system. Science. 2014;343: 80–4. doi: 10.1126/science.1246981 24336569 10.1126/science.1246981 PMC3972032

[12] Holkers M, Maggio I, Henriques SFD, Janssen JM, Cathomen T, Gonçalves M a F V. Adenoviral vector DNA for accurate genome editing with engineered nucleases. Nat Methods. 2014; doi: 10.1038/nmeth.3075 25152084 10.1038/nmeth.3075

[13] Ratz M, Testa I, Hell SW, Jakobs S. CRISPR/Cas9-mediated endogenous protein tagging for RESOLFT super-resolution microscopy of living human cells. Sci Rep. 2015;5: 9592 doi: 10.1038/srep09592 25892259 10.1038/srep09592 PMC4402611

[14] Schwank G, Koo B-K, Sasselli V, Dekkers JF, Heo I, Demircan T, et al Functional repair of CFTR by CRISPR/Cas9 in intestinal stem cell organoids of cystic fibrosis patients. Cell Stem Cell. 2013;13: 653–8. doi: 10.1016/j.stem.2013.11.002 24315439 10.1016/j.stem.2013.11.002

[15] Kabadi a. M, Ousterout DG, Hilton IB, Gersbach C a. Multiplex CRISPR/Cas9-based genome engineering from a single lentiviral vector. Nucleic Acids Res. 2014;42: gku749–. doi: 10.1093/nar/gku749 25122746 10.1093/nar/gku749 PMC4231726

[16] Li C, Guan X, Du T, Jin W, Wu B, Liu Y, et al Inhibition of HIV-1 infection of primary CD4+ T cells by gene editing of CCR5 using adenovirus-delivered CRISPR/Cas9. J Gen Virol. 2015; vir.0.000139–. doi: 10.1099/vir.0.000139 25854553 10.1099/vir.0.000139

[17] Abrahimi P, Chang WG, Kluger MS, Qyang Y, Tellides G, Saltzman WM, et al Efficient Gene Disruption in Cultured Primary Human Endothelial Cells by CRISPR/Cas9. Circ Res. 2015;117: 121–8. doi: 10.1161/CIRCRESAHA.117.306290 25940550 10.1161/CIRCRESAHA.117.306290 PMC4490936

[18] Claussnitzer M, Dankel SN, Kim K-H, Quon G, Meuleman W, Haugen C, et al FTO Obesity Variant Circuitry and Adipocyte Browning in Humans. N Engl J Med. 2015;373: 150819140043007 doi: 10.1056/NEJMoa1502214 26287746 10.1056/NEJMoa1502214 PMC4959911

[19] Bellec J, Bacchetta M, Losa D, Anegon I, Chanson M, Nguyen TH. CFTR Inactivation by Lentiviral Vector-mediated RNA Interference and CRISPR-Cas9 Genome Editing in Human Airway Epithelial Cells. Curr Gene Ther. 2015;15: 447–59. Available: http://www.ncbi.nlm.nih.gov/pubmed/26264708 26264708 10.2174/1566523215666150812115939

[20] Van Wetering S, van der Linden AC, van Sterkenburg MA, de Boer WI, Kuijpers AL, Schalkwijk J, et al Regulation of SLPI and elafin release from bronchial epithelial cells by neutrophil defensins. Am J Physiol Lung Cell Mol Physiol. 2000;278: L51–L58. 10645890 10.1152/ajplung.2000.278.1.L51

[21] Arts GJ, Langemeijer E, Tissingh R, Ma L, Pavliska H, Dokic K, et al Adenoviral vectors expressing siRNAs for discovery and validation of gene function. Genome Res. 2003;13: 2325–2332. doi: 10.1101/gr.1332603 12975310 10.1101/gr.1332603 PMC403715

[22] Michiels F, van Es H, van Rompaey L, Merchiers P, Francken B, Pittois K, et al Arrayed adenoviral expression libraries for functional screening. Nat Biotechnol. 2002;20: 1154–1157. doi: 10.1038/nbt746 12355097 10.1038/nbt746

[23] Rippmann JF, Schoelch C, Nolte T, Pavliska H, van Marle A, van Es H, et al Improved lipid profile through liver-specific knockdown of liver X receptor alpha in KKAy diabetic mice. J Lipid Res. 2009;50: 22–31. doi: 10.1194/jlr.M700571-JLR200 18769020 10.1194/jlr.M700571-JLR200

[24] Cong L, Ran F, Cox D, Lin S, Barretto R. Multiplex Genome Engineering Using CRISPR / Cas Systems. Science (80-). 2013;819 doi: 10.1038/nbt1319 10.1126/science.1231143 PMC3795411 23287718

[25] Ten Dijke P, Arthur HM. Extracellular control of TGFbeta signalling in vascular development and disease. Nat Rev Mol Cell Biol. 2007;8: 857–869. doi: 10.1038/nrm2262 17895899 10.1038/nrm2262

[26] Popp MW, Maquat LE. Leveraging rules of nonsense-mediated mRNA decay for genome engineering and personalized medicine. Cell. 2016 pp. 1319–1332. doi: 10.1016/j.cell.2016.05.053 27259145 10.1016/j.cell.2016.05.053 PMC4924582

[27] N. Lekkerkerker A, Aarbiou J, van Es T, A. J. Janssen R. Cellular Players in Lung Fibrosis. Current Pharmaceutical Design. 2012 pp. 4093–4102. doi: 10.2174/138161212802430396 22630084 10.2174/138161212802430396

[28] Hu B, Wu Z, Phan SH. Smad3 mediates transforming growth factor-beta-induced alpha-smooth muscle actin expression. Am J Respir Cell Mol Biol. 2003;29: 397–404. doi: 10.1165/rcmb.2003-0063OC 12702545 10.1165/rcmb.2003-0063OC

[29] Ghosh a K, Yuan W, Mori Y, Varga J. Smad-dependent stimulation of type I collagen gene expression in human skin fibroblasts by TGF-beta involves functional cooperation with p300/CBP transcriptional coactivators. Oncogene. 2000;19: 3546–3555. doi: 10.1038/sj.onc.1203693 10918613 10.1038/sj.onc.1203693

[30] Gasiunas G, Barrangou R, Horvath P, Siksnys V. PNAS Plus: Cas9-crRNA ribonucleoprotein complex mediates specific DNA cleavage for adaptive immunity in bacteria. Proc Natl Acad Sci. 2012;109: E2579–E2586. doi: 10.1073/pnas.1208507109 22949671 10.1073/pnas.1208507109 PMC3465414

[31] Sternberg SH, Doudna JA. Expanding the Biologist’s Toolkit with CRISPR-Cas9. Mol Cell. Elsevier Inc.; 2015;58: 568–574. doi: 10.1016/j.molcel.2015.02.032 26000842 10.1016/j.molcel.2015.02.032

[32] Friedland AE, Tzur YB, Esvelt KM, Colaiácovo MP, Church GM, Calarco JA. Heritable genome editing in C. elegans via a CRISPR-Cas9 system. Nat Methods. 2013;10: 741–3. doi: 10.1038/nmeth.2532 23817069 10.1038/nmeth.2532 PMC3822328

[33] Bassett AR, Tibbit C, Ponting CP, Liu J-L. Highly efficient targeted mutagenesis of Drosophila with the CRISPR/Cas9 system. Cell Rep. 2013;4: 220–8. doi: 10.1016/j.celrep.2013.06.020 23827738 10.1016/j.celrep.2013.06.020 PMC3714591

[34] Hwang WY, Fu Y, Reyon D, Maeder ML, Tsai SQ, Sander JD, et al Efficient genome editing in zebrafish using a CRISPR-Cas system. Nat Biotechnol. 2013;31: 227–9. doi: 10.1038/nbt.2501 23360964 10.1038/nbt.2501 PMC3686313

[35] Wang H, Yang H, Shivalila CS, Dawlaty MM, Cheng AW, Zhang F, et al One-step generation of mice carrying mutations in multiple genes by CRISPR/Cas-mediated genome engineering. Cell. 2013;153: 910–8. doi: 10.1016/j.cell.2013.04.025 23643243 10.1016/j.cell.2013.04.025 PMC3969854

[36] Niu Y, Shen B, Cui Y, Chen Y, Wang J, Wang L, et al Generation of gene-modified cynomolgus monkey via Cas9/RNA-mediated gene targeting in one-cell embryos. Cell. 2014;156: 836–43. doi: 10.1016/j.cell.2014.01.027 24486104 10.1016/j.cell.2014.01.027

[37] Tan W, Carlson DF, Lancto CA, Garbe JR, Webster DA, Hackett PB, et al Efficient nonmeiotic allele introgression in livestock using custom endonucleases. Proc Natl Acad Sci U S A. 2013;110: 16526–31. doi: 10.1073/pnas.1310478110 24014591 10.1073/pnas.1310478110 PMC3799378

[38] Platt RJ, Chen S, Zhou Y, Yim MJ, Swiech L, Kempton HR, et al CRISPR-Cas9 Knockin Mice for Genome Editing and Cancer Modeling. Cell. 2014;159: 440–55. doi: 10.1016/j.cell.2014.09.014 25263330 10.1016/j.cell.2014.09.014 PMC4265475

[39] Maggio I, Holkers M, Liu J, Janssen JM, Chen X, Gonçalves M a F V. Adenoviral vector delivery of RNA-guided CRISPR/Cas9 nuclease complexes induces targeted mutagenesis in a diverse array of human cells. Sci Rep. 2014;4: 5105 doi: 10.1038/srep05105 24870050 10.1038/srep05105 PMC4037712

[40] Chu HW, Rios C, Huang C, Wesolowska-Andersen A, Burchard EG, O’Connor BP, et al CRISPR-Cas9 mediated gene knockout in primary human airway epithelial cells reveals a pro-inflammatory role for MUC18. Gene Ther. 2015; doi: 10.1038/gt.2015.53 26043872 10.1038/gt.2015.53 PMC4600011

[41] Fu Y, Foden J a, Khayter C, Maeder ML, Reyon D, Joung JK, et al High-frequency off-target mutagenesis induced by CRISPR-Cas nucleases in human cells. Nat Biotechnol. Nature Publishing Group; 2013;31: 822–6. doi: 10.1038/nbt.2623 23792628 10.1038/nbt.2623 PMC3773023

[42] Mali P, Aach J, Stranges PB, Esvelt KM, Moosburner M, Kosuri S, et al CAS9 transcriptional activators for target specificity screening and paired nickases for cooperative genome engineering. Nat Biotechnol. 2013;31: 833–8. doi: 10.1038/nbt.2675 23907171 10.1038/nbt.2675 PMC3818127

[43] Veres A, Gosis BS, Ding Q, Collins R, Ragavendran A, Brand H, et al Low incidence of Off-target mutations in individual CRISPR-Cas9 and TALEN targeted human stem cell clones detected by whole-genome sequencing. Cell Stem Cell. 2014;15: 27–30. doi: 10.1016/j.stem.2014.04.020 24996167 10.1016/j.stem.2014.04.020 PMC4082799

[44] Wu X, Scott D a, Kriz AJ, Chiu AC, Hsu PD, Dadon DB, et al Genome-wide binding of the CRISPR endonuclease Cas9 in mammalian cells. Nat Biotechnol. 2014;32: 670–676. doi: 10.1038/nbt.2889 24752079 10.1038/nbt.2889 PMC4145672

[45] Kuscu C, Arslan S, Singh R, Thorpe J, Adli M. Genome-wide analysis reveals characteristics of off-target sites bound by the Cas9 endonuclease. Nat Biotechnol. 2014;32: 677–683. doi: 10.1038/nbt.2916 24837660 10.1038/nbt.2916

