# Supplementary figures and images for: Highly efficient gene inactivation by adenoviral CRISPR/Cas9 in human primary cells
Source: PLoS One. 2017 Aug 11;12(8):e0182974. doi: 10.1371/journal.pone.0182974 (PMC5553774; doi:10.1371/journal.pone.0182974)

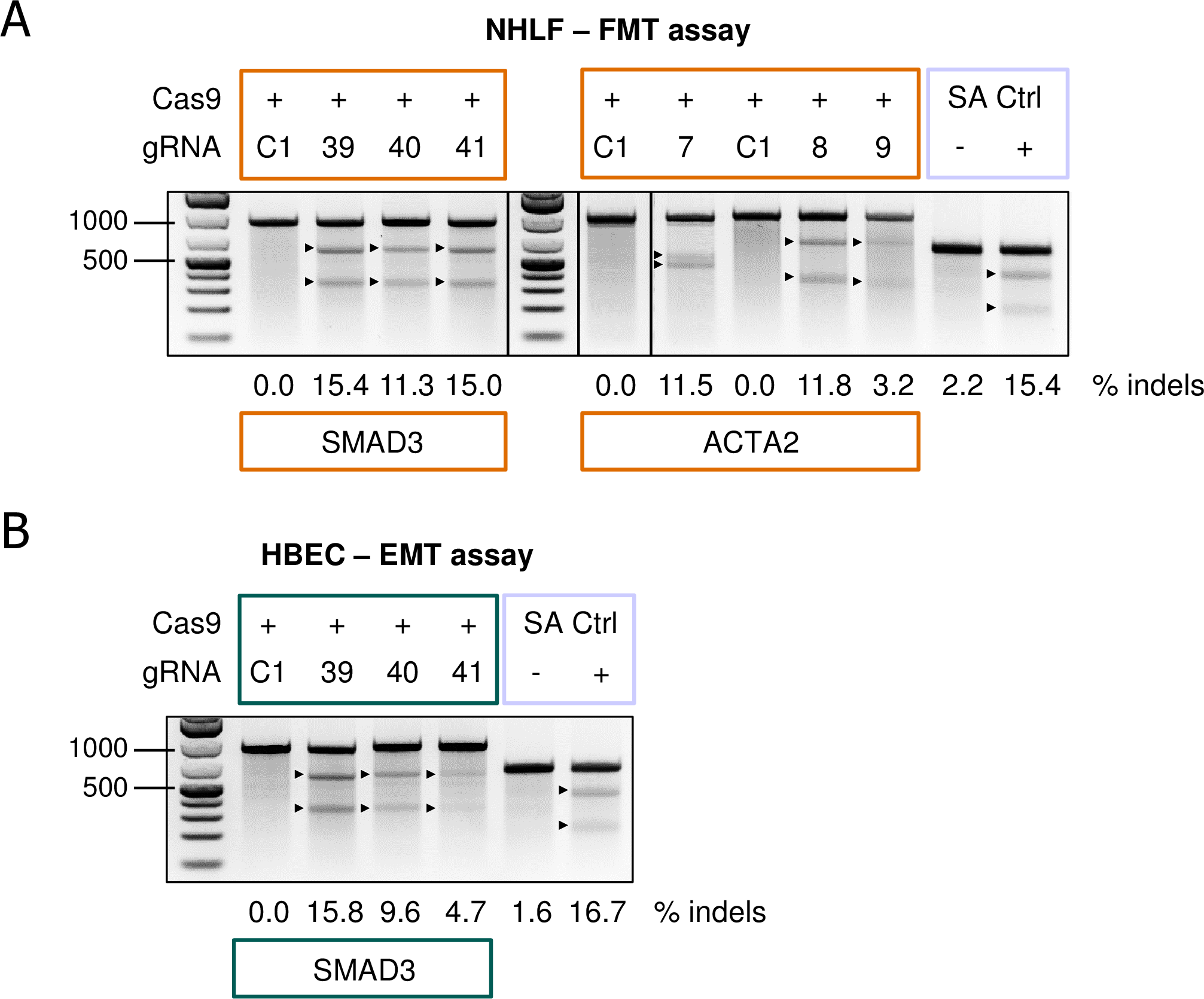

Supplement: S1 Fig — (A) Genomic editing of the SMAD3 and ACTA2 genes in NHLFs co-transduced with Cas9 and SMAD3- or ACTA2-targeting gRNA AdV. Cells were co-transduced at DIV 1 at total MOI 30 (Cas9:gRNA ratio 1:2), followed by addition of TGF-β1 at DIV 6. Genomic DNA was analyzed for the presence of indels at DIV 9 by SURVEYOR® assay as described earlier. Black arrowheads mark the DNA fragments that appear after successful gene editing. Indel frequencies are shown below each lane and were determined by densitometry of the full-length and cleaved PCR fragments as described above. (B) Genomic editing of the SMAD3 gene in HBECs co-transduced with Cas9 and SMAD3-targeting gRNA AdV. Cells were co-transduced at total MOI 12 (Cas9:gRNA ratio 1:1) followed by EMT induced by addition of a cocktail containing TGF-β1 and TNFα at DIV 6. Genomic DNA was analyzed for the presence of indels at DIV 9 by SURVEYOR® assay as described earlier. 39, 40, 41: gRNAs targeting SMAD3; 7, 8, 9: gRNAs targeting ACTA2; C1: gRNA targeting control sequence; SA Ctrl: SURVEYOR® assay control. (TIFF) [file pone.0182974.s001.tiff]

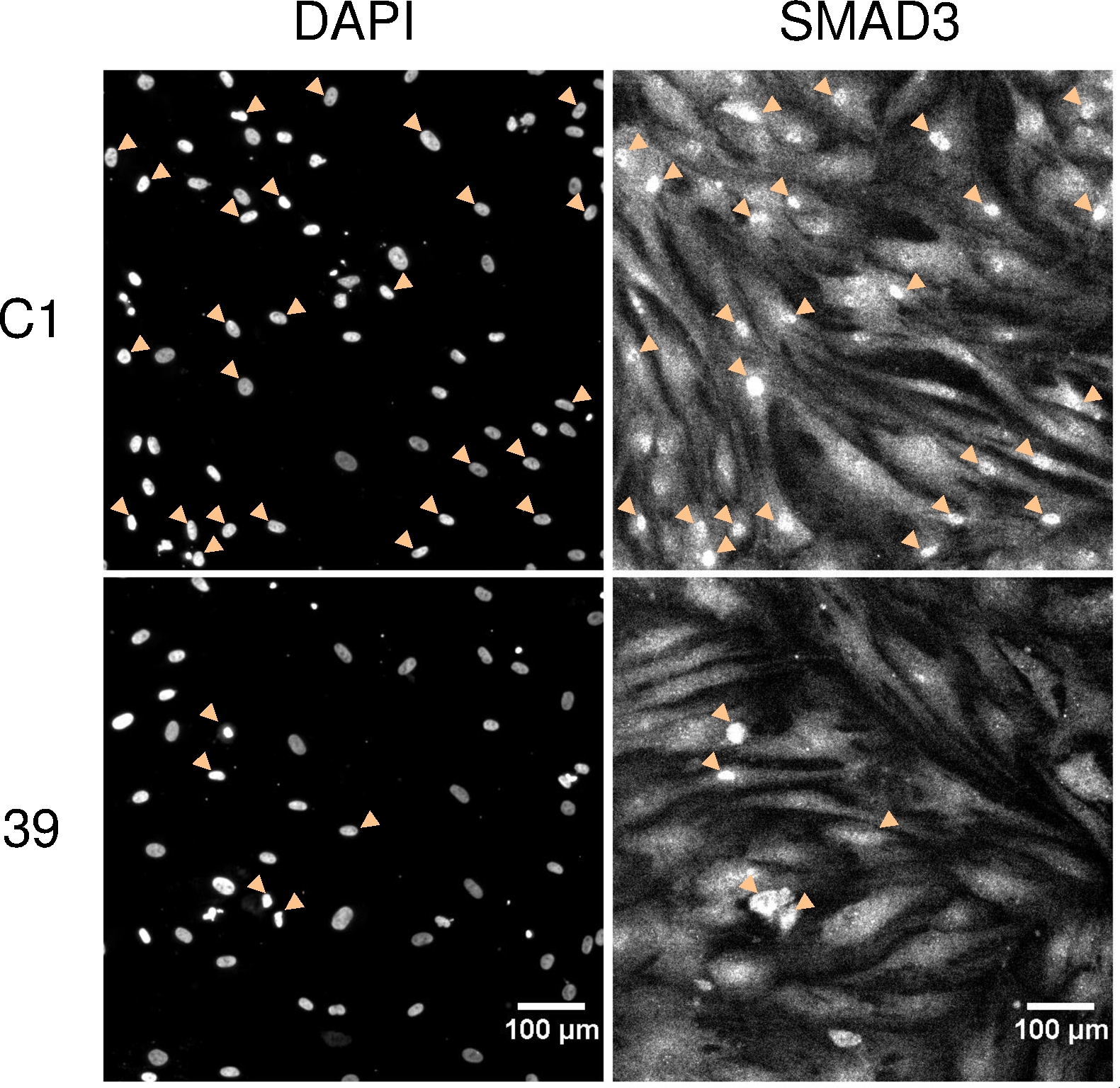

Supplement: S2 Fig — Visualization of SMAD3 nuclear translocation in NHLFs co-transduced with Cas9 and SMAD3-targeting gRNA AdV. Primary NHLFs were co-transduced at DIV 1 at total MOI 30 (Cas9:gRNA ratio 1:2), followed by addition of TGF-β1 at DIV 6. Cells were fixed for immunofluorescent labelling of SMAD3 and nuclear DAPI staining. Exemplifying images of SMAD3 and nuclear DAPI labelling are shown. Translocated SMAD3 is highlighted with orange arrowheads. 39: gRNA targeting SMAD3; C1: gRNA targeting control sequence. (TIF) [file pone.0182974.s002.tif]

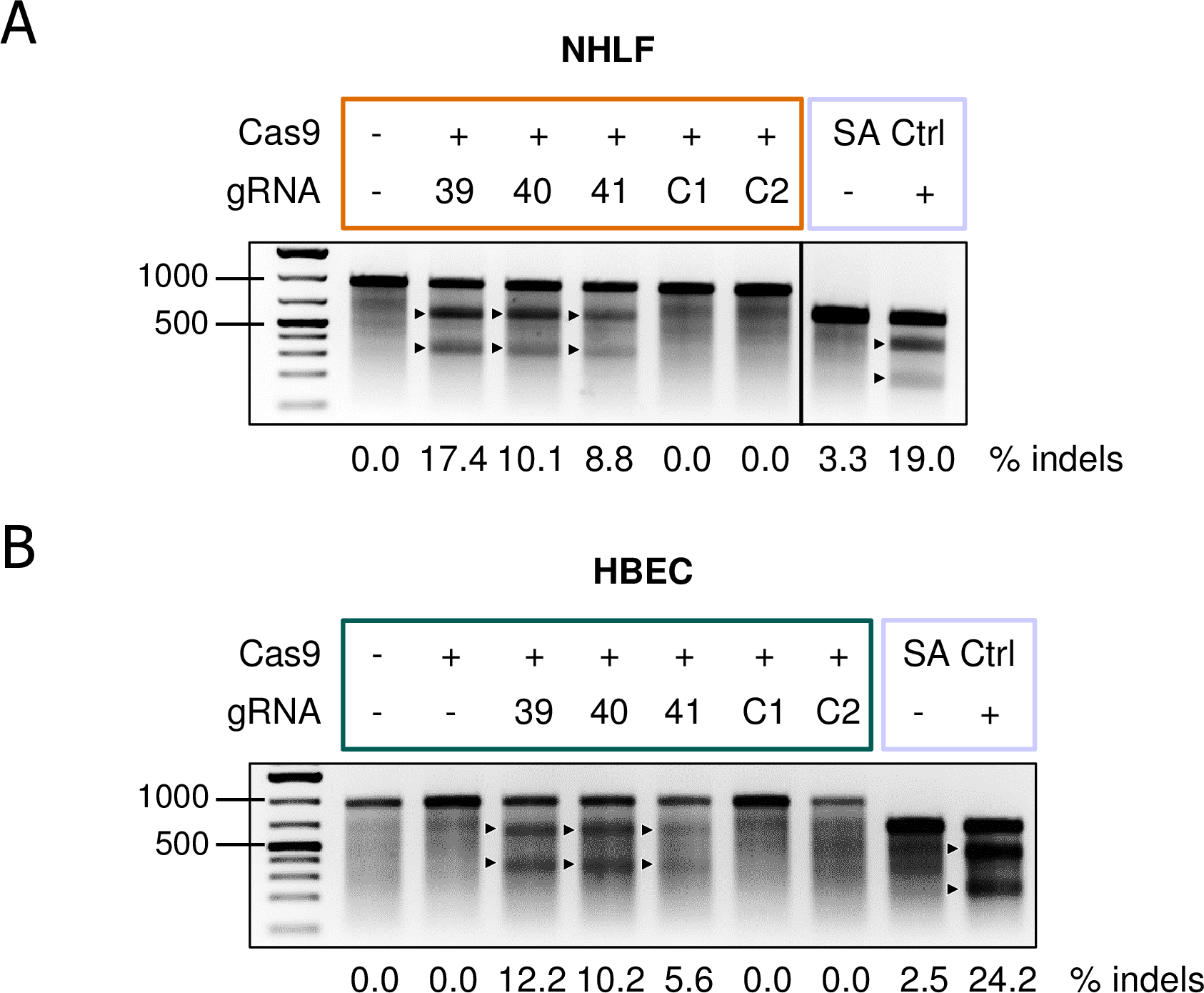

Supplement: S3 Fig — (A) Genomic editing of the SMAD3 gene in NHLFs following co-TD with Cas9 and SMAD3-targeting gRNA AdV. NHLFs were co-transduced at DIV 1 at total MOI 30 (Cas9:gRNA ratio 1:2) followed by genomic DNA isolation at DIV 7 and PCR amplification of the SMAD3 target region. Resulting PCR products were used for SURVEYOR® assay analysis and resolved by agarose gel electrophoresis as described earlier. Indel frequencies are shown below each lane determined by densitometry of the full-length and cleaved PCR fragments (black arrowheads). (B) Genomic editing of the SMAD3 gene in HBECs co-transduced with Cas9 and SMAD3-targeting gRNA AdV. Primary HBECs were co-transduced as above, but with a total MOI 14 (Cas9:gRNA ratio 1:2.5). Genomic DNA was analyzed for the presence of indels at DIV 4 by SURVEYOR® assay as described above. 39, 40, 41: gRNAs targeting SMAD3; C1, C2: gRNAs targeting control sequences; SA Ctrl: SURVEYOR® assay control. (TIFF) [file pone.0182974.s003.tiff]

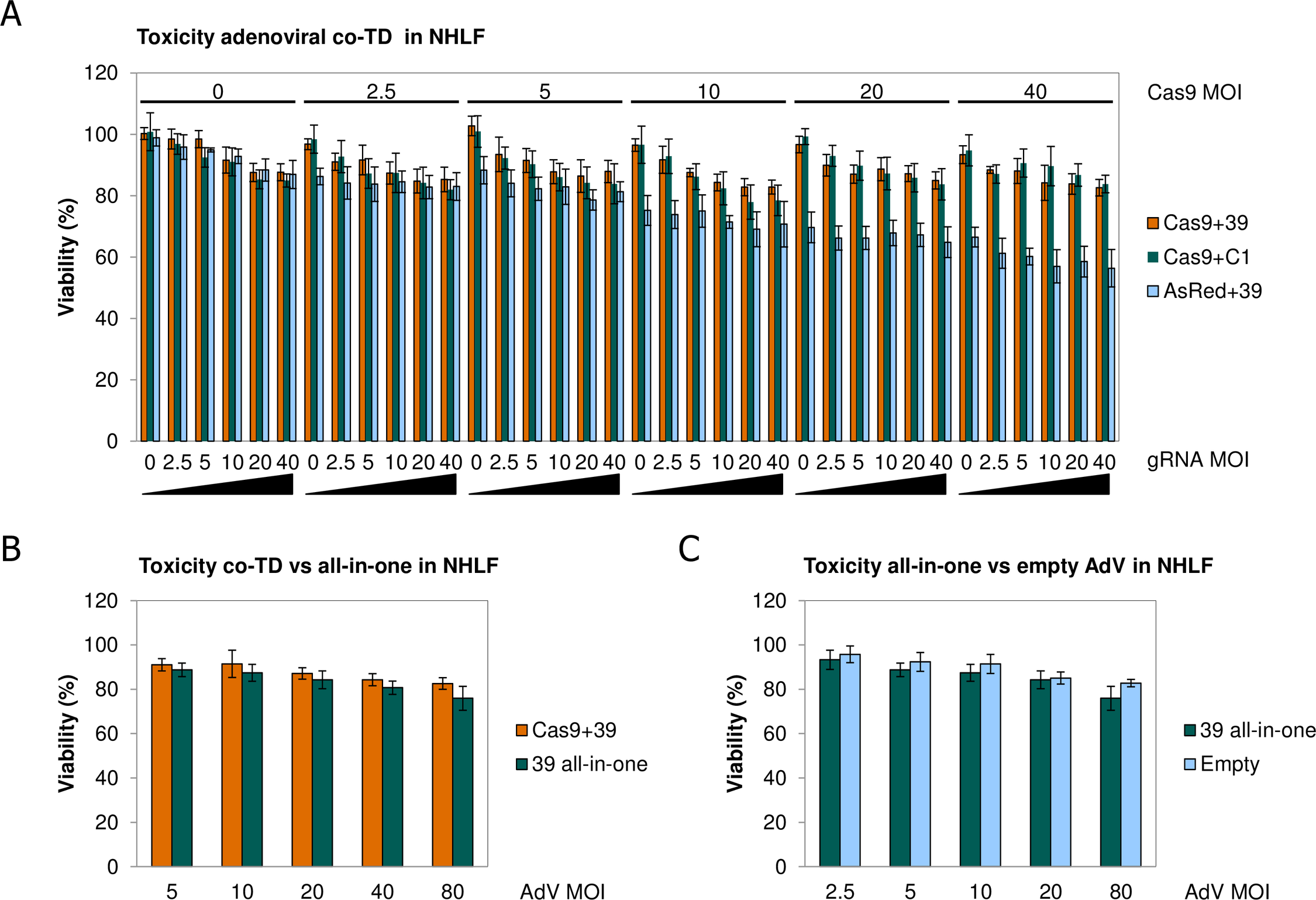

Supplement: S4 Fig — Assessment of toxicity resulting from TD of NHLFs with varying amounts of AdV. (A) Primary NHLFs were co-transduced at DIV 1 with varying ratios of Cas9 and gRNA AdV or AsRed and gRNA AdV at a maximal total MOI of 80 followed by addition of TGF-β1 at DIV 6. Cell viability was determined at DIV 9 by using the CellTiter-Blue® Cell Viability Assay. Data are normalized to the untreated condition. (B) NHLFs were treated as in A, except that cells were either co-transduced or transduced with the single all-in-one AdV. For the co-TD approach only the data from Cas9:gRNA AdV ratio of 1:1 are utilized for comparison with the all-in-one system. (C) NHLFs were treated as in A, except that cells were transduced with a single all-in-one Cas9/gRNA AdV or an AdV particle without insert (“empty”). For the comparison of co-TD (ratio 1:1) with the all-in-one approach, it is important to note that equal MOIs will result in double molar amounts of Cas9 and gRNA with the all-in-one AdV, as the total MOI is kept constant. Data points are from quadruplicate wells (n = 4) and error bars represent standard deviation. (TIFF) [file pone.0182974.s004.tiff]

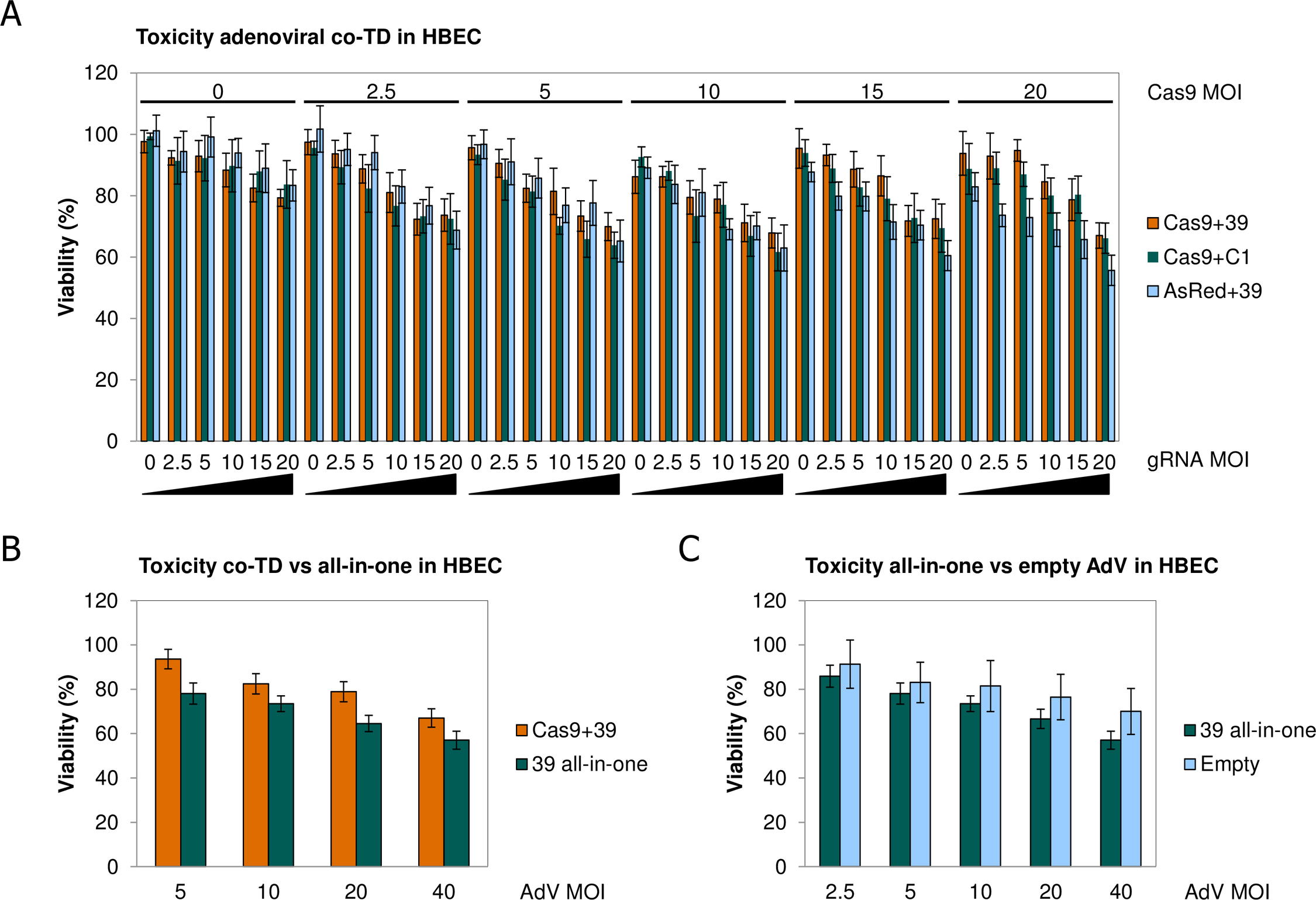

Supplement: S5 Fig — Assessment of toxicity resulting from TD of HBECs with varying amounts of AdV. (A) Primary HBECs were co-transduced at DIV 1 with varying ratios of Cas9 and gRNA AdV or AsRed and gRNA AdV at a maximal total MOI of 40 followed by addition of a cocktail containing TGF-β1 and TNFα at DIV 6. Cell viability was determined at DIV 9 by using the CellTiter-Blue® Cell Viability Assay. Data are normalized to the untreated condition. (B) HBECs were treated as in A, except that cells were either co-transduced or transduced with the single all-in-one AdV. For the co-TD approach only the data from Cas9:gRNA AdV ratio of 1:1 are utilized for comparison with the all-in-one system. (C) HBECs were treated as in A, except that cells were transduced with a single all-in-one Cas9/gRNA AdV or an AdV particle without insert (“empty”). For the comparison of co-TD (ratio 1:1) with the all-in-one approach, it is important to note that equal MOIs will result in double molar amounts of Cas9 and gRNA with the all-in-one AdV, as the total MOI is kept constant. Data points are from quadruplicate wells (n = 4) and error bars represent standard deviation. (TIFF) [file pone.0182974.s005.tiff]

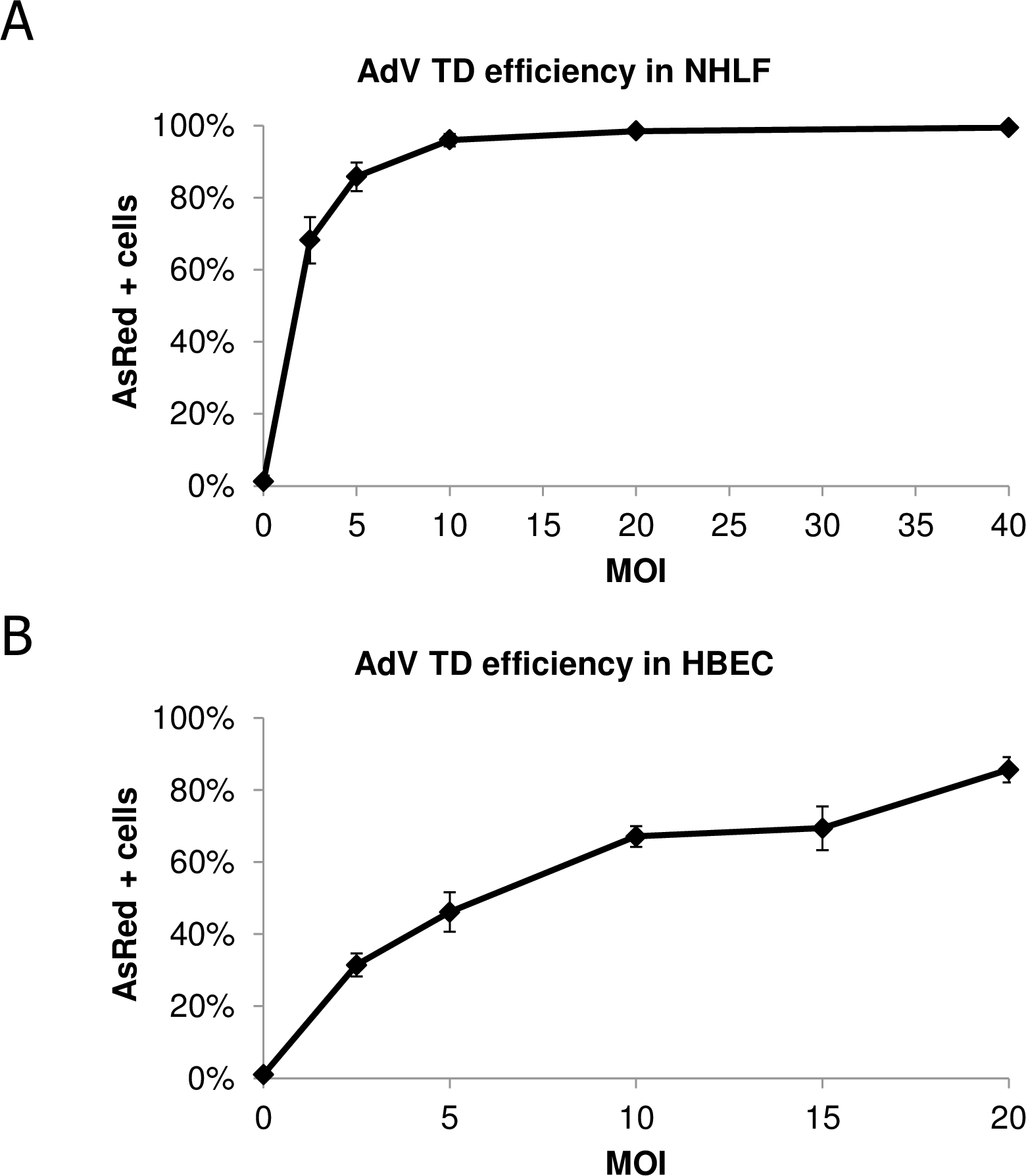

Supplement: S6 Fig — Determination of TD efficiencies using an AsRed reporter-gene AdV. (A) NHLFs were transduced at DIV 1 with an AsRed AdV at various MOIs ranging from 2.5–40, after which cells were fixed and stained with DAPI at DIV 9. Cells were imaged to determine the number of viable, AsRed-positive cells by HCA (see Materials and Methods for details). (B) HBECs were transduced at DIV 1 with an AsRed AdV at various MOIs ranging from 2.5–20 at DIV 1, after which cells were fixed and stained with DAPI at DIV 9. Cells were imaged to determine the number of viable, AsRed-positive cells by HCA as above (see Materials and Methods for details). Data points are from six biological replicates (n = 6 wells) and error bars represent standard deviation. (TIFF) [file pone.0182974.s006.tiff]

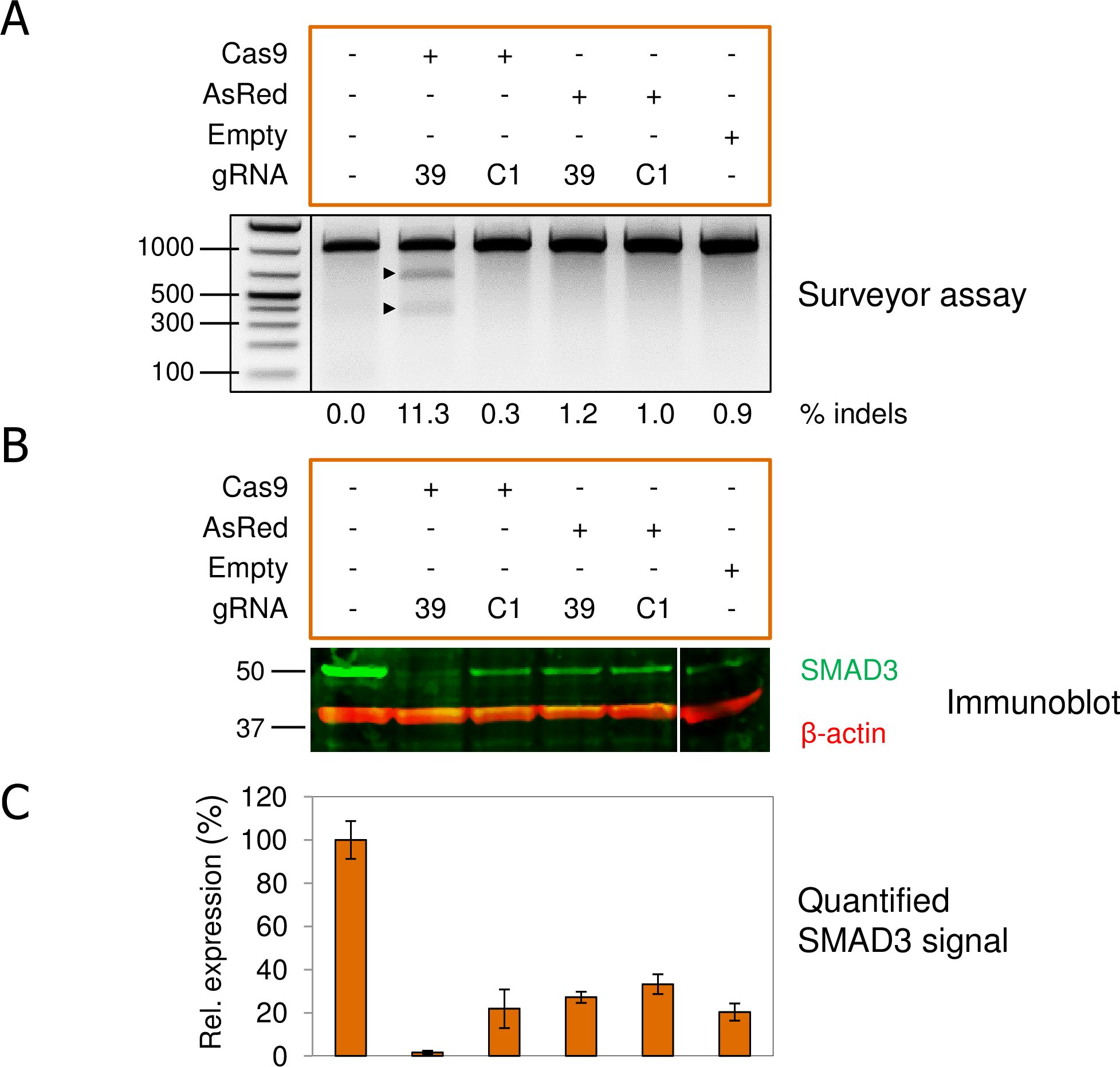

Supplement: S7 Fig — (A) SMAD3 genomic editing in NHLFs following co-TD with Cas9 or AsRed and gRNA AdV or single TD with an “empty” AdV particle (no insert). Primary NHLFs were transduced at total MOI 30 (Cas9:gRNA ratio 1:2) after which genomic DNA was analyzed for the presence of indels at DIV 8 by SURVEYOR® assay as described earlier. (B) AdV CRISPR/Cas9-mediated SMAD3 protein KD in NHLFs. Cells were harvested at DIV 8 for protein analysis by immunoblotting. A total of 30–35 μg protein per lane was loaded. Immunoblots were stained with the indicated primary antibodies. (C) Quantification of SMAD3 protein levels by densitometry of the bands depicted in panel B. Expression is blotted relative to the untreated condition and normalized to the β-actin signal. Data points are from biological duplicate samples (n = 2) and error bars represent standard deviation. (TIFF) [file pone.0182974.s007.tiff]

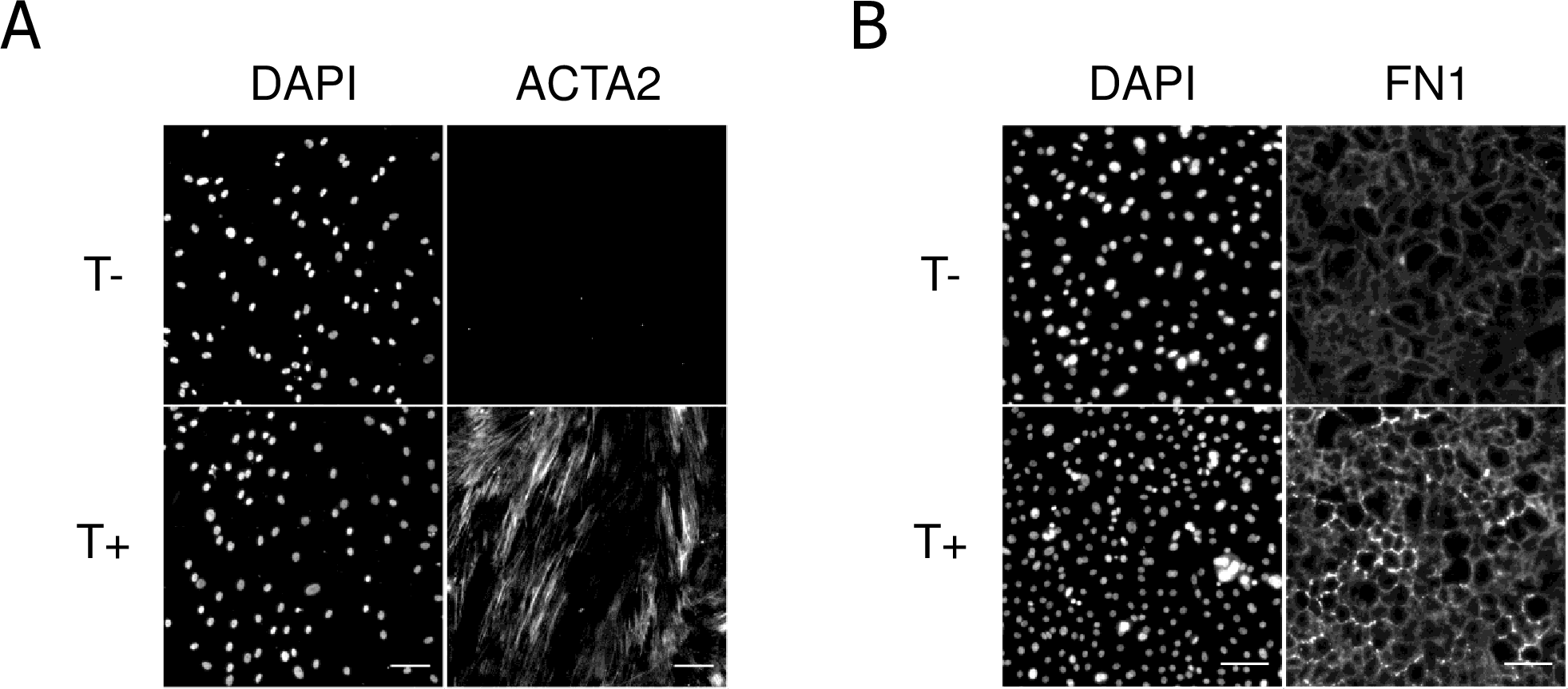

Supplement: S8 Fig — (A) NHLFs were triggered at DIV 6 with TGF-β1 to induce FMT and fixed at DIV 9 followed by immunofluorescent labelling of ACTA2 and DAPI nuclear staining. Representative images of the triggered (T+) and untriggered (T-) conditions are shown. (B) HBECs were triggered at DIV 6 with a cocktail containing TGF-β1 and TNFα to induce EMT and fixed at DIV 9 followed by immunofluorescent labelling of FN1 and DAPI nuclear staining. Representative images of the triggered (T+) and untriggered (T-) conditions are shown. Scale bars indicate 100 μm. (TIFF) [file pone.0182974.s008.tiff]

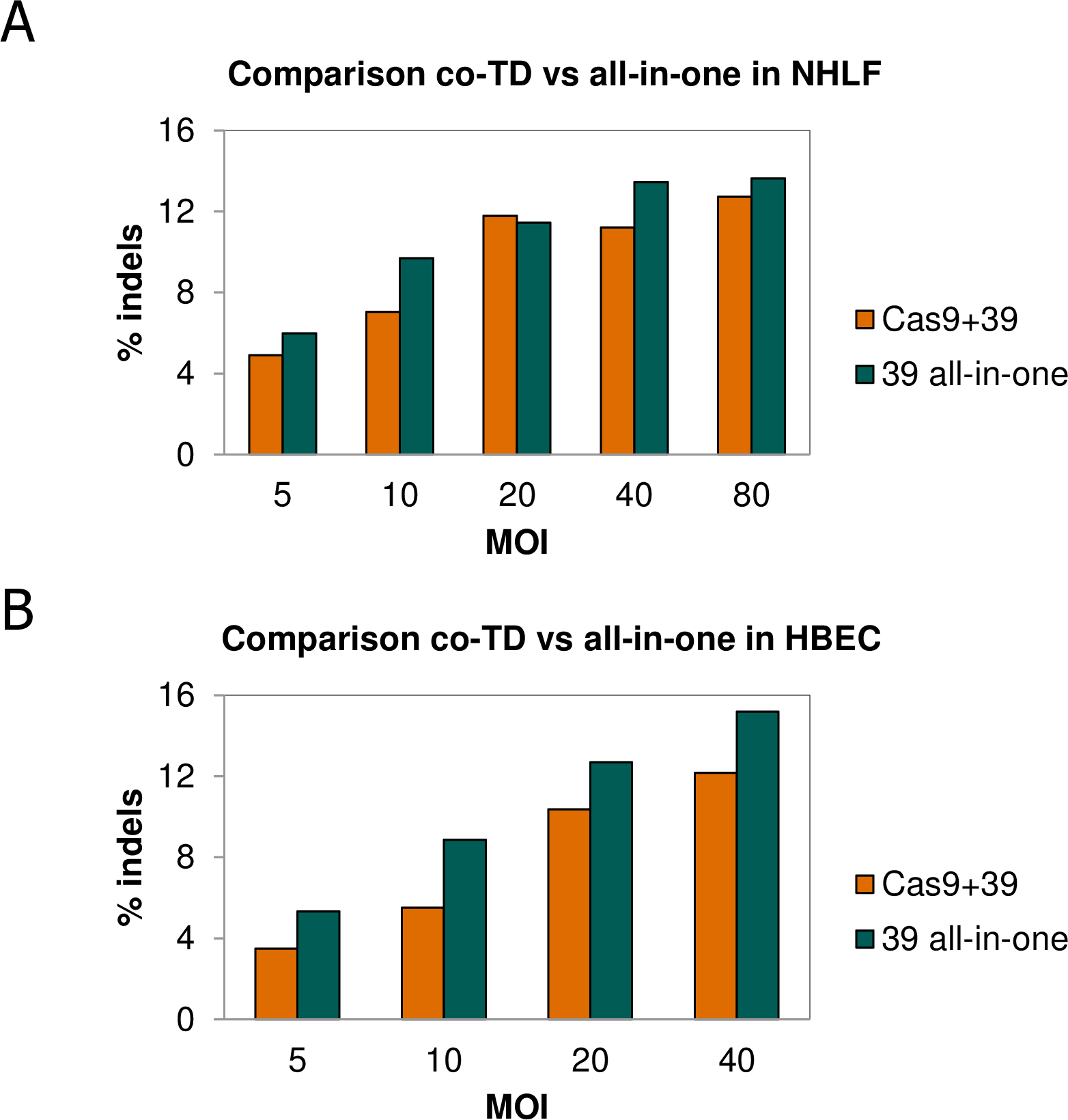

Supplement: S9 Fig — (A) Primary NHLFs were either co-transduced or transduced with an all-in-one AdV at DIV 1 at a maximal total MOI of 80. In case cells were co-transduced with Cas9 and gRNA AdV the ratio of Cas9:gRNA was 1:1 to allow for direct comparison with the all-in-one AdV. NHLFs were harvested at DIV 9 after which genomic DNA was analyzed for the presence of indels by SURVEYOR® assay as described earlier. Indel frequencies were quantified as determined by densitometry of the DNA fragments depicted in Fig 2A and 2C. (B) Primary HBECs were treated as in A, except that TD was performed at a maximal total MOI of 40. Indel frequencies were quantified as determined by densitometry of the DNA fragments depicted in Fig 3A and 3C. Cas9+39: co-TD with Cas9 and SMAD3 targeting gRNA; 39 all-in-one: TD with a SMAD3 targeting all-in-one AdV. (TIFF) [file pone.0182974.s009.tiff]
