# Supplementary material for: Highly efficient gene inactivation by adenoviral CRISPR/Cas9 in human primary cells
Source: PLoS One. 2017 Aug 11;12(8):e0182974. doi: 10.1371/journal.pone.0182974 (PMC5553774; doi:10.1371/journal.pone.0182974)
Supplement: S1 Table — The expected PCR fragments after successful genomic editing detected by SURVEYOR® assay are shown. All gRNAs are preceded by a guanine nucleotide (underlined) at the 5’ end to promote transcription driven by the U6 promoter. NA: not applicable. (DOCX) [file pone.0182974.s010.docx]

**S1 Table. Primer and gRNA sequences used in this study.**

| **Primer sequence (5'-3')** | **Target** | **gRNA ID** | **gRNA sequence (5'-3')** | **SURVEYOR^®^** **fragment (bp)** | | |
| --- | --- | --- | --- | --- | --- | --- |
|  |  |  |  | **1** | **2** | **3** |
| TGCTGAGTTGCAGGGTTTCT | SMAD3 | SMAD3_v39 (39) | GGGACAUCGGAUUCGGGGAU | 933 | 593 | 340 |
| CTGACCACAGACCCTCGTTC |  |  |  |  |  |  |
| TGCTGAGTTGCAGGGTTTCT | SMAD3 | SMAD3_v40 (40) | GGCUGGGGACAUCGGAUUCG | 933 | 598 | 335 |
| CTGACCACAGACCCTCGTTC |  |  |  |  |  |  |
| TGCTGAGTTGCAGGGTTTCT | SMAD3 | SMAD3_v41 (41) | GUGCUGGGGACAUCGGAUUC | 933 | 599 | 334 |
| CTGACCACAGACCCTCGTTC |  |  |  |  |  |  |
| TGCTGAGTTGCAGGGTTTCT | SMAD3 | eGFP_v9 (C1) | GGCCACAAGUUCAGCGUGUC | 933 | - | - |
| CTGACCACAGACCCTCGTTC |  |  |  |  |  |  |
| TGCTGAGTTGCAGGGTTTCT | SMAD3 | eGFP_v11 (C2) | GAAGGGCAUCGACUUCAAGG | 933 | - | - |
| CTGACCACAGACCCTCGTTC |  |  |  |  |  |  |
| TCTGGATTTCCCATCTGTGCTC | ACTA2 | ACTA2_v7 (7) | GUGGGACGUCCCACAAUGGA | 1001 | 539 | 462 |
| TACCATGTGATGCCACCAATACT |  |  |  |  |  |  |
| CACCGCTGAAACCTTCTGGA | ACTA2 | ACTA2_v8 (8) | GGAGUUACGAGUUGCCUGAU | 1051 | 686 | 365 |
| TTGCTACCCTTTCCGCTGTC |  |  |  |  |  |  |
| CACCGCTGAAACCTTCTGGA | ACTA2 | ACTA2_v9 (9) | GCGGAAACGUUCAUUUCCGA | 1051 | 708 | 343 |
| TTGCTACCCTTTCCGCTGTC |  |  |  |  |  |  |
| ACACCTGATCAAGCCTGTTCATTTGATTAC | NA | NA (SA Ctrl) | NA | 633 | 416 | 217 |
| CGCCAAAGAATGATCTGCGGAGCTT |  |  |  |  |  |  |

The expected PCR fragments after successful genomic editing detected by SURVEYOR^®^ assay are shown. All gRNAs are preceded by a guanine nucleotide (underlined) at the 5’ end to promote transcription driven by the U6 promoter. NA: not applicable.
